# A general approach to protein folding using thermostable exoshells

**DOI:** 10.1038/s41467-021-25996-4

**Published:** 2021-09-29

**Authors:** Samira Sadeghi, Siddharth Deshpande, Girish Vallerinteavide Mavelli, Alphan Aksoyoglu, Jayesh Bafna, Mathias Winterhalter, R. Manjunatha Kini, David P. Lane, Chester L. Drum

**Affiliations:** 1 Yong Loo Lin School of Medicine, National University of Singapore, Singapore, Singapore; 2 Genome Institute of Singapore (GIS), Agency for Science, Technology and Research (A*STAR), Singapore, Singapore; 3 Life Science and Health, Jacobs University Bremen, Bremen, 28759 Germany; 4 Department of Biological Science, Faculty of Science, National University of Singapore, Singapore, Singapore; 5 P53 Laboratory, Agency for Science, Technology and Research (A*STAR), Singapore, Singapore

**Keywords:** Nanoparticles, Chaperones

## Abstract

In vitro protein folding is a complex process which often results in protein aggregation, low yields and low specific activity. Here we report the use of nanoscale exoshells (tES) to provide complementary nanoenvironments for the folding and release of 12 highly diverse protein substrates ranging from small protein toxins to human albumin, a dimeric protein (alkaline phosphatase), a trimeric ion channel (Omp2a) and the tetrameric tumor suppressor, p53. These proteins represent a unique diversity in size, volume, disulfide linkages, isoelectric point and multi versus monomeric nature of their functional units. Protein encapsulation within tES increased crude soluble yield (3-fold to >100-fold), functional yield (2-fold to >100-fold) and specific activity (3-fold to >100-fold) for all the proteins tested. The average soluble yield was 6.5 mg/100 mg of tES with charge complementation between the tES internal cavity and the protein substrate being the primary determinant of functional folding. Our results confirm the importance of nanoscale electrostatic effects and provide a solution for folding proteins in vitro.

## Abstract (web summary)

In vitro protein folding can often result in aggregation and low yields. Here the authors use nanoscale exoshells to improve soluble yield, functional yield and specific activity of folded proteins.

## Introduction

Efficient in vitro folding of proteins has been a goal of biochemistry since the early experiments of Anfinsen, which demonstrated the relationship of primary sequence to native structure^1^. Despite the advantages of producing functional proteins without the need of complex and potentially contaminating expression hosts, solutions for in vitro protein folding currently remain complex and often result in an insoluble or improperly folded product^2^. Chaperone proteins, which help to promote folding and reduce aggregation in vivo, have been suggested as a solution to the problems of in vitro folding, however, the potential use of chaperone proteins remains rare as they are difficult to produce at scale, have limited effect sizes, and are often characterized for only a single, or very few, protein substrates^3^. In a compendium of 1046 in vitro folding protocols (RefoldDB), only 6 protocols (0.5%) incorporate an added chaperone for protein folding^4^.

Chaperone proteins use long-range electrostatic interactions, short-range hydrophobic forces, and the physical confinement of protein substrates within a molecular cage to promote protein folding^5,6^. In the setting of recombinant protein expression, coexpression of GroEL/ES (a large multimeric ATPase that can encapsulate unfolded proteins within its internal volume) or DnaJ/K (a classic heat-shock protein that binds unfolded surfaces of target proteins) can enhance soluble yield^7^. The generalizability of this approach remains a challenge, however, as the improvement in cytoplasmic solubility is protein-specific and coexpression can be toxic to the expression host^8–11^.

Recombinantly expressed natural chaperones have also been used to assist in vitro folding protocols^12^, however, significant issues remain including in vitro inactivation^13^, difficulty in scale-up^14^ and size- and charge-based restrictions to a small number of protein substrates^3,15^. Likewise, an additional stripping reagent is often required to separate the protein substrate from the added chaperone, which may denature the native substrate^12,16^.

Chemical chaperones such as glycerol, trehalose, and various lipid species are also used in in vitro protein folding^17^. However, effect sizes are often low and frequent optimization and monitoring are required to ensure preservation of the native protein structure and retention of biological activity^18,19^. Thus, there remains a need for improved platform technologies that can be applied across protein classes with relative technical ease.

**Fig. 1:**
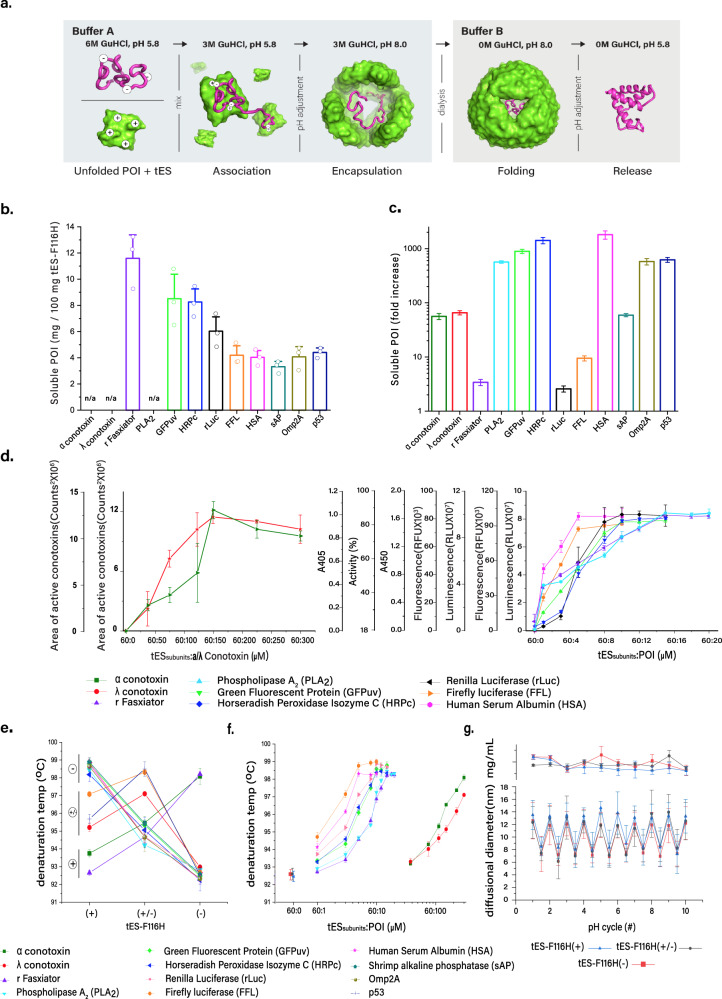
Comparisons of tES–F116H folding. **a** Description of the tES nanoencapsulation protocol depicting buffer adjustments and buffer exchange via dialysis (**b**) Crude soluble yields of POI increase after tES–F116H nanoencapsulation. As the amount of tES added to the folding mix was the limiting factor for POI-soluble yield, the results are expressed as mg POI/100 mg of tES–F116H. **c** Fold increase of soluble POI folded with tES–F116H compared with its absence. **d** Functional yields of POI are determined by the ratio of POI to charge complementary tES–F116H_subunits_ in the folding mix and demonstrate saturation with titration. **e** Charge complementation of the tES–F116H interior with POI determines stabilizing energetics. Three tES–F116H charge variants are assayed and POI self-organizes according to their charge (labeled next to the ordinate) and stabilizing effect. **f** Low-molecular-weight proteins require high molar ratios to stabilize the assembly. Thermal denaturation temperatures of tES–F116H were measured via DFS as the concentation of POI is titrated against tES–F116H during the loading phase of the folding protocol. **g** tES–F116H undergo pH-mediated assembly and disassembly. Shell diameter can be monitored using DLS (lower panel) and no significant soluble protein loss is observed after 10 cycles (upper panel). (**b**–**g**, Data are presented as mean ± SEM., *n* = 3 independent experiments) (Source data are provided as a Source Data file).

We hypothesize that engineered nanoscale shells, which recapitulate many of the properties of natural chaperones but have the scalability and stability of a reagent, could be used to promote the in vitro folding of a wide range of proteins of interest (POI). Prior studies have indicated that macromolecules covalently fused to the interior of the ferritin gene (*afuFer*) from *Archeoglobus fulgides* can fold and be protected from external denaturants, albeit with low yields^20^. In this study, we engineered a general approach to noncovalently encapsulate, fold, promote disulfide pairing, and release functionally active proteins of interest at suitable scale (mg/L culture) for laboratory use (Fig. 1a; supplementary Fig. 1). Compared with the use of fusion proteins, a noncovalent system offers (i) far greater yields of POI (~5 mg/100 mg of tES versus <5 µg/100 mg of tES for the fusion-protein approach), (ii) improved quality of folded product (i.e., specific activity) compared with non-tES-mediated folding, and (iii) a protocol simple enough to be used in general laboratory practice.

## Results

### tES shells

tES shells are 24-mers based on a truncation mutant of the native ferritin sequence from *Archaeoglobus fulgides*. tES monomers contain histidine substitutions at sites of 3-fold symmetry (F116H), which result in pH-titratable assembly and disassembly of the shell. To provide charge complementation of client proteins, tES charge variants have net positive [tES–F116H(+)], negative [tES–F116H(–)], and neutral [tES-F116H(+/–)] interior charges^20^. tES subunits were expressed individually and mixed with POI as indicated in supplementary Fig. 1.

All three variants of tES can be expressed (≥400 mg/L shaking-flask cell culture), purified (≥90%), and concentrated (200 mg/mL) using standard techniques. Two POIs (conotoxins) were synthesized and ten proteins (supplementary Fig. 2) were individually expressed as denatured inclusion bodies in *E. coli* to serve as a panel to characterize the effects of tES. Purified tES–F116H shells were mildly acidified for disassembly (pH < 6.0), following which the subunits were separated in size-exclusion chromatography (SEC). POI samples in solubilization buffers were incubated in the presence of tES subunits at different ratios, until the optimum tES–F116H_subunits_:POI ratio was reached for maximum encapsulation efficiency. The pH of the mixture was adjusted to 8.0 followed by overnight dialysis in refolding buffer (supplementary Fig. 1). The folded protein was purified using SEC and checked for its activity. POI alone was chosen as the control and subjected to identical conditions in all cases.

### Small protein toxins

**Fig. 2:**
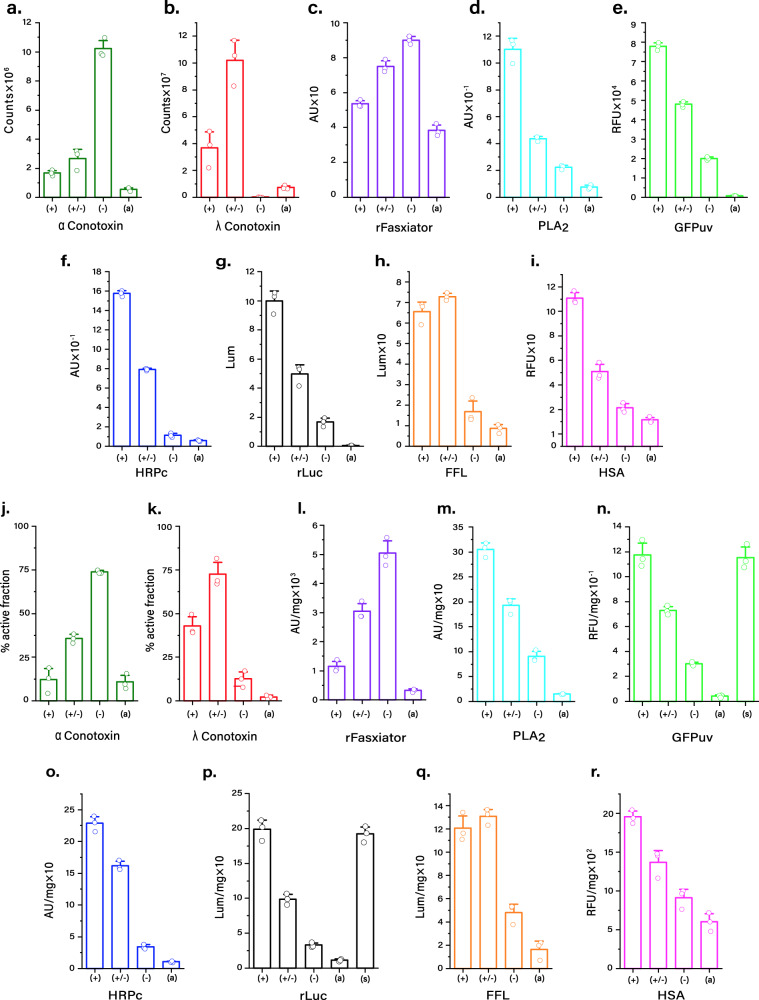
Effect of tES–F116H on POI activity. **a**–**i** Activity assays of POI after folding with tES–F116H charge variants and in the absence of tES–F116H. **j**–**r** Specific activity is increased with tES–F116H folding when compared with the respective POI-only folding. tES–F116H(+), tES–F116H(+/–), tES–F116H(–) and POI alone are indicated as (+), (+/–), (–), and (a), respectively. GFPuv and rLuc could also be expressed as a soluble protein in the same expression; thus, POI purified from this soluble fraction is indicated as (s) and used as positive control. The molar ratio for each POI was 150:60 (Conotoxins), 15:60 (rFasxiator/PLA_2_), 10:60 (HRP/GFP/rLuc), and 5:60 (HSA/FFL). (Data are presented as mean ± SEM., *n* = 3 independent experiments) (Source data are provided as a Source Data file).

The diverse effects of small peptide toxins have contributed to fundamental advances in neuroscience in addition to the design of approved therapeutics^21^. Aggregation and structural heterogeneity often prevent the folding of synthetic peptide toxins in vitro^22,23^. The effect of tES was tested with three structurally distinct toxins. α-Conotoxin (ImI P6 amide, 1.35 kDa) is a 12-amino-acid peptide with a net positive surface charge. *α*-Conotoxin can form either a bioactive conformation (C1–3/C2–4) or bioinactive conformations, depending on the disulfide pairing of four cysteine residues (C1–4/C2–3 or C1–2/C3–4). *λ*-Conotoxin (CMrVIA K6, 1.24 kDa) has 10 amino acids, a net neutral charge, and likewise can fold into bioactive (C1–4/C2–3) or bioinactive (C1–3/C2–4 and C1–2/C3–4) conformations based on the disulfide pairing of four cysteines^23^. When the tES charge complemented the net charge of the peptide, i.e., tES–F116H(–) for *α*-conotoxin and tES–F116H( + /–) for *λ*-conotoxin, tES increased crude soluble yield (60-fold for both), functional yield (10-fold for both), and specific activity (6-fold for *α*-conotoxin and 23-fold for *λ*-conotoxin) (Figs. 1c, d; 2a, b, j and k; supplementary Figs. 3, 4).

**Fig. 3:**
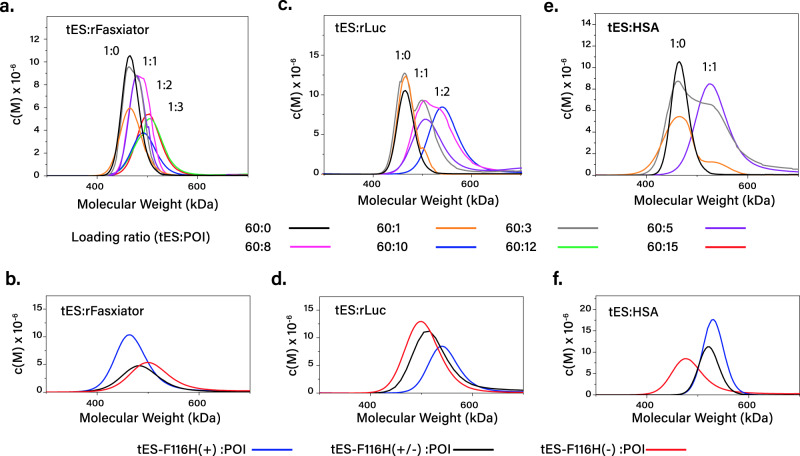
Monomeric POI encapsulation using tES–F116H. **a** tES–F116H can encapsulate three rFasxiator molecules (36 kDa, total, loading ratio of 60:15) (AUC peaks are labeled with the number of encapsulated POI with colors corresponding to the molar ratios of titrated POI during the folding protocol). **b** tES–F116H charge is a critical determinant of encapsulation efficiency. Positively charged rFasxiator exhibits no encapsulation in tES–F116H(+). **c** Two rLuc molecules (72 kDa, total, loading ratio of 60:10) can be accommodated in tES–F116H. **d** Negatively charged rLuc shows optimal encapsulation in tES–F116H(+). **e** tES accomodates one HSA (66 kDa, total, loading ratio of 60:5) molecule. **f** Negatively charged HSA shows optimal encapsulation in tES–F116H(+).

rFasxiator (12 kDa) is a Kunitz‐type inhibitor of factor XIa, has a net positive charge, and three disulfide bridges^24^. The use of tES–F116H(–) with rFasxiator increased crude soluble yield (3-fold), functional yield (3-fold), and specific activity (12-fold) (Figs. 1b–d; 2c, l; supplementary Figure 5a; Figure supplementary Fig. 6a). The encapsulation ratio was measured using analytical ultracentrifugation (AUC), which demonstrated three rFasxiator molecules encapsulated per tES–F116H(–), whereas the same charge tES–H116(+) showed no encapsulation (Fig. 3a, b).

### Phospholipase A_2_

Acidic phospholipase A_2_ (PLA_2_, 17 kDa) is a calcium-dependent enzyme found in snake venom (*Agkistrodon halyspallas*), which cleaves fatty acids from phospholipids, resulting in arachidonic acid release and localized inflammation^25^. PLA_2_ is negatively charged, contains seven conserved disulfide bridges, and no prior in vitro folding studies have been reported, whereas extensive precipitation was noted in the POI-only refolding mix. The addition of tES–F116H(+) increased crude soluble yield (500-fold), functional yield (13-fold), and specific activity (20-fold) of PLA_2_ (Figs. 1b–d; 2d, m; supplementary Fig. 5b).

### Green fluorescent protein

Green fluorescent protein (GFPuv, 27 kDa) is a negatively charged model protein for folding studies and undergoes highly variable expression in *E.coli*, dependent on induction conditions^26,27^; thus, GFPuv overexpression in *E. coli* results in both soluble and insoluble fractions. Correct folding of the native GFPuv structure can be inferred from the development of 508-nm fluorescence, which requires autocatalytic formation of the fluorophore and a surrounding *β*-barrel structure. tES–F116H( + ) folding of GFPuv inclusion bodies increased crude soluble yield (900-fold), functional yield (30-fold), and specific activity (30-fold). GFPuv purified from the soluble fraction of *E. coli* exhibited comparable specific activity (0.8-fold) to tES–F116H( + ) folded GFPuv (Figs. 1b–d; 2e, n; supplementary Fig. 5c).

### Horseradish peroxidase

HRP is an industrial enzyme used in chromogenic assays, however, it requires production from the root of the horseradish plant (*Armoracia rusticana*), resulting in seasonal yield variability and a heterogeneous mixture of isoenzymes^28^. We studied recombinant expression of HRP isozyme C (HRPc, 34 kDa), a truncated version of HRP, which forms inactive inclusion bodies in *E. coli*. Refolding of HRPc requires the presence of calcium, formation of four disulfide bonds, the incorporation of a heme prosthetic group, and currently results in low yields (2–3%) of active enzyme^29^. tES–F116H(+) increased crude soluble yield (1400-fold), functional yield (18-fold), and specific activity (20-fold) of HRPc (Figs. 1b–d; 2f, o; supplementary Fig. 5d).

### Renilla luciferase

Renilla luciferin 2-monooxygenase (rLuc, 36 kDa) is a negatively charged, easily denatured intracellular protein that catalyzes the production of blue light in the presence of oxygen and coelenterazine substrate^30^. Factors that affect the folding of rLuc are unknown as in vitro folding of rLuc, which has not been previously reported. tES–F116H(+) increased crude soluble yield (3-fold), functional yield (80-fold), and specific activity (25-fold) (Figs. 1b–d; 2g, p; supplementary Fig. 5e; Fig. supplementary Fig. 6b). Using AUC, we found a ratio of two rLuc molecules to each tES–F116H(+). tES–F116H(+) demonstrated maximal encapsulation compared with tES–F116H(+/–) and tES–F116H(–), with a consistent peak broadening corresponding to an additional population of tES with a single rLuc (Fig. 3c, d)

### Firefly luciferase

Firefly luciferase (FFL, 60 kDa) is a negatively charged bioluminescent protein widely used in bioassays that oxidizes luciferin substrate in the presence of ATP, Mg^2+^ ion, and oxygen. FFL serves as a model for the study of ATPase enzymes; however, folding and stability of FFL remains a significant challenge^31^. The structural fold of FFL is not related to rLuc and consists of two lobular domains that exist in either an extended conformation (which would exceed the internal diameter of the tES cage) or a compact conformation with substrate bound at the domain interface^30^. tES–F116H(+/–) increased crude soluble yield (10-fold), functional yield (8-fold) and specific activity (7-fold) of FFL (Figs. 1b–d; 2h, q; supplementary Fig. 5f).

### Human albumin

Human serum albumin (HSA, 66 kDa) is the most abundant protein in blood and is an important infusional therapeutic. Recombinant expression and in vitro folding protocols for HSA have proven difficult and HSA currently requires purification from human blood products^32^. HSA is a globular protein with three α-helical domains and seventeen disulfide bonds and which complicates in vitro folding due to the formation of inappropriate interdomain pairing^33^. tES–F116(+) increased crude soluble yield (1800-fold), functional yield (12-fold), and specific activity (3-fold) of HSA (Figs. 1b–d; 2i, r; supplementary Fig. 5g; supplementary Fig. 6c). Using AUC, we found a ratio of a single HSA molecule to each tES–F116H(+), whereas tES–F116H(+/–) and tES–F116H(–) encapsulate either not at all or with very low frequency (Fig. 3e, f).

### In vitro folding of multimeric proteins

After our study of monomeric interactions with tES, we then tested the hypothesis that multimeric protein assemblies could be productively folded. About 30–50% of all proteins exist natively as homomultimers, yet only a few solutions exist for in vitro folding of multimeric proteins^34^. The natural assembly of protein multimers is thought to occur sequentially. Starting with a structured monomer, which then associates with other subunits to form, after further conformational changes, a multimeric assembly. Structured monomers are inherently unstable and often rely on chaperones to produce the appropriate fold required for quaternary assembly^35^. We hypothesized that structural monomers could be folded, transiently stabilized, and, upon release from tES–F116H nanoparticle, mutually assemble without aggregation in vitro.

### Alkaline phosphatase, a dimeric enzyme

**Fig. 4:**
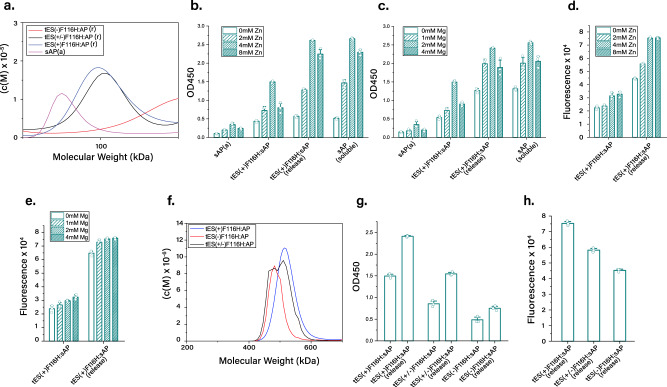
Alkaline phosphatase folding with tES–F116H. **a** Release of sAP from tES–F116H charge variants results in a dimeric peak for tES–F116H(+) and tES–F116H(+/–), however, monomeric sAP was observed with sAP folded alone [sAP(a)] and aggregation was seen with the use of tES-F116H(–). **b**, **c** Titration of Zn and Mg yields activity increases similar for caged versus released sAP and a solubly expressed sAP control. **d, e** Titration of Zn and Mg has differential effects on sAP intrinsic fluorescence, a marker of folding. **f** AUC of tES-F116H(+) loaded with sAP demonstrates a M.W. of 522 kDa, consistent with encapsulation of a single sAP monomer. **g** Activity of sAP is moderately increased after release from three charge variants of tES–F116H. **h** Intrinsic fluorescence is optimal when sAP is folded with charge-matched tES–F116H(+). (**b**, **c**, **d**, **e**, **g**, **h**, data are presented as mean ± SEM., *n* = 3 independent experiments) (Source data are provided as a Source Data file).

Alkaline phosphatases (AP) are negatively charged homodimeric metalloenzymes that contain two intrachain disulfide bonds and are used widely in both industry and research^36^. An exception to this is a monomeric form of AP isolated from the Vibrio genus^37^. Shrimp AP (sAP, 52 kDa) is active at low temperatures (0–15 °C) and denatures above 30 °C, resulting in its extensive use for cloning protocols^38^. The thermolability of sAP makes it a challenge for in vitro folding and no in vitro folding protocols have been published to date. APs have been reported to form numerous intermediate proteins during both unfolding and refolding processes^39^ and when sAP is folded in vitro without tES, it aggregates and fails to form dimers (Fig. 4a). Consistent with a hypothesis of structured monomer assembly, the use of charge-complementary tES to fold sAP was critical for sAP dimer formation (Fig. 4a). The role of zinc and magnesium ions in sAP function and folding was likewise studied and consistent with prior reports^40,41^. tES–F116H(+) crude soluble yield (60-fold), increased functional yield (9-fold) and specific activity (10-fold) of sAP (Figs. 1b, c; 4b–e). No dimer formation was observed when sAP was folded in the absence of tES (Fig. 4a).

Because the steric limit of tES enforces monomeric encapsulation, isolated subunits of multimeric enzymes can be studied in solution. The active site of AP does not require catalytic residues contributed from a paired dimer and thermolabile, monomeric isoforms of AP can be isolated from cold-adapted Vibrio species^42^. After confirming monomeric encapsulation via AUC (Fig. 4f) and isolating tES–F116H:sAP complexes, we found remarkable activity of the sAP monomer while it was encapsulated in the tES–F116H(+) cage and only a 2-fold activity increase after release from tES (Fig. 4g, h). This stands in contrast to a study of *E. coli* AP, which reported a 10,000-fold difference in monomeric versus dimeric activity^43^. Consistent with our findings, Olsen et al. have reported sAP to be a homodimer in solution, however, observed in situ catalytic activity for sAP monomers separated via native gel electrophoresis^44^. The specific activity of sAP dimer released from tES was equivalent to a positive control, whereas activity of encapsulated sAP monomers was reduced by ~50% (Fig. 4b, c).

### Omp2A, a trimeric ion channel

**Fig. 5:**
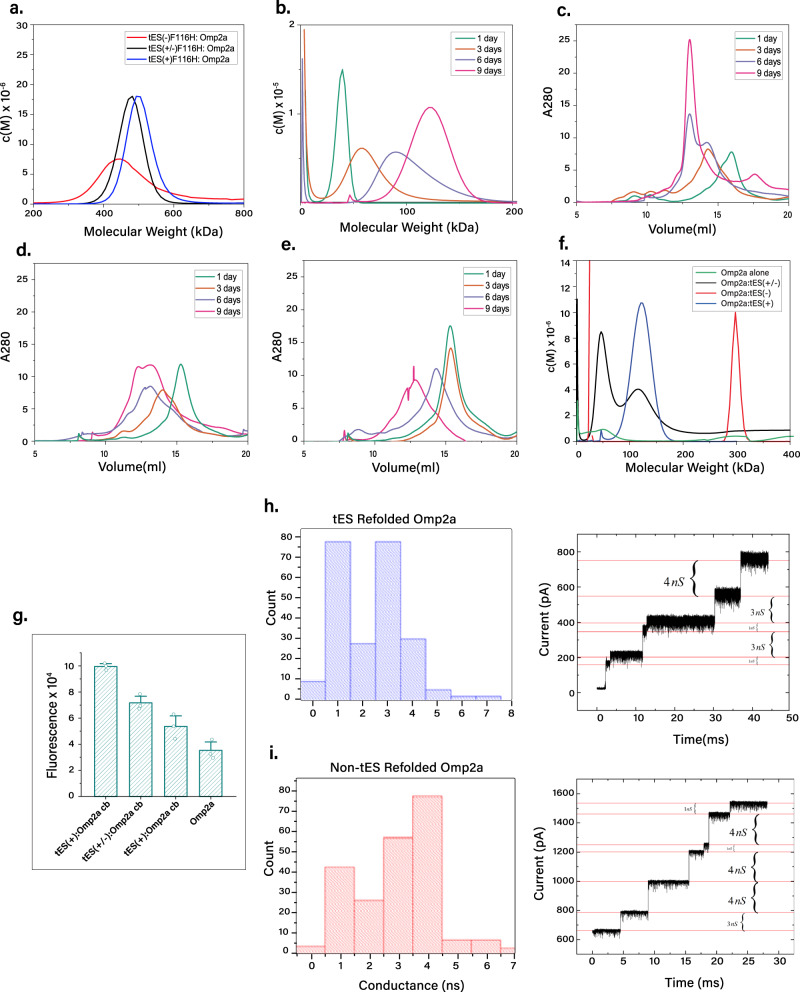
Omp2a folding with tES–F116H. **a** tES–F116H(+) encapsulates a single monomer of Omp2a (**b**) Omp2a monomers released from tES–F116H(+) form trimeric assemblies after a nine-day incubation period. **c**, **d**, **e** SEC demonstrates complete trimerization only in the setting of tES116H(+)-mediated folding. **f** AUC demonstrates that the ability to form Omp2a trimers is affected by tES–F116H internal charge. Omp2a folded without tES–F116H does not demonstrate trimerization. **g** Intrinsic fluorescence of Omp2a refolded and released from tES(+)F116H, tES(+/–)F116H, and tES(–)F116H, and Omp2a refolded without tES. (Data are presented as mean ± SEM., *n* = 3 independent experiments). **h** Conductance measurements of the 172-kDa Omp2a trimeric SEC fraction demonstrate a range of 1–4 nS. **i** A positive control of Omp2a demonstrates similar conductance insertions (Source data are provided as a Source Data file).

Porins are homotrimeric ion channels natively expressed in the outer membrane of bacteria, mitochondria, and chloroplasts. Omp2a (39 kDa) is a negatively charged porin isolated from *Brucella melitensis*, studied for its biophysical properties and as an antigen for the prevention of brucellosis. Omp2a is toxic when overexpressed as a soluble protein and is recombinantly expressed as an inclusion body^45^. Production of Omp2a, and porins in general, thus remains a significant challenge for studies of channel function. We performed nanoencapsulation using three tES charge variants on Omp2a and, using AUC, found a shift in M.W. (Fig. 5a), consistent with a single encapsulated monomer per either tES–F116H(+) or tES–F116H(+/–) shell. No encapsulation was seen with tES–F116H(–) (Fig. 5a). Following the nanoencapsulation protocol and substrate release, Omp2a subunits were subjected to a 9-day incubation period at 37 °C (Fig. 5b). Size profiles were assessed via sequential sampling and SEC analysis (Fig. 5c–e). AUC confirmed that only Omp2a subunits released from tES–F116H(+) formed a monodisperse peak at 170 kDa, consistent with complete trimerization (Fig. 5f). When Omp2a was folded within tES–F116H(+/–), it partially multimerized into trimers after nine days, however, when Omp2a was folded under identical conditions without tES, or using tES–F116H(–), no evidence of trimer assembly was seen on SEC or AUC (Fig. 5a, f).

Tryptophan fluorescence is a measure of Omp2a folding^45^ and was increased following Omp2a trimerization (Fig. 5g). To confirm ion channel activity of the assembled trimers, the tES–F116H(+)-folded Omp2a 170-kDa fraction was studied in membrane bilayers. In a prior study, Omp2a protein was found to be unstable, conductances were highly variable (~45 pS–650 pS), and no trimer activity could be observed^46^. In our study, Omp2a trimers demonstrated conductances of 3 nS or 4 nS with occasional conductances of 1 nS (Fig. 5h). As no electrophysiological characterizations of Omp2a trimer formations have been reported for comparison, we used the method of Roussel et al. to obtain small amounts of Omp2a trimers^47,48^ and no significant differences in conductance or gating were observed (Fig. 5i). The gating and conductance of trimeric Omp2a thus appears to be similar to its trimeric homolog, Omp2b^46^. tES–F116H(+) increased crude soluble yield (900-fold), functional yield (>100-fold), and specific activity (>100-fold) of Omp2 (Figs. 1b, c; 5h).

### p53, a tetrameric tumor suppressor

Tumor protein 53 (p53, 43.7 kDa) is a homotetrameric tumor suppressor and the most commonly mutated gene (>50%) in human cancers^49^. Tetramerization of p53 monomers is essential for biological activity and heteromultimerization with p53 mutants can account for the differential penetrance of common cancer mutations^50^. Post-translational modifications of p53 are complex and regulate multiple cellular functions, including apoptosis, senescence, and response to physiological stress^51^. For the present study, we have used a stabilized p53 clone that forms functional tetramers in vitro and is used for the in vitro study of p53 as a regulatory node^52^.

**Fig. 6:**
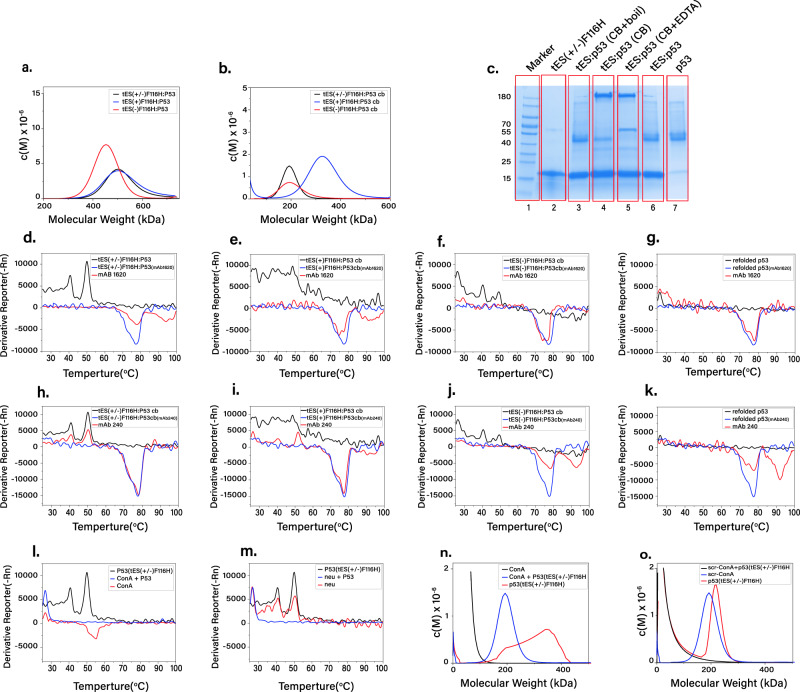
p53 folding and tetramerization using tES–F116H. **a** AUC demonstrates tES–F116H(+/−) and tES–F116H(+) encapsulated a single p53 monomer. **b** p53 release from tES–F116H(+/−) and tES–F116H(+) results in tetramer formation as demonstrated by AUC. The use of tES–F116H(–) results in aggregation. **c** SDS gel demonstrates monomeric p53 in the tES:POI complex, and tetrameric p53 after monomer release. Lane 2 shows the tES subunits. Prior to tES release, p53 monomer and tES–F116H(+/−) subunits were mixed (pH 8.0) and visualized using SDS-PAGE as a 47 kDa and 20 kDa (lane 6). After tES release, p53 was predominantly seen as an ~180-kDa band that was attributed to the tetramer (lane 4). Upon denaturation (boiling with reducing reagents) of the same sample, the tetramer band disappeared and a 47-kDa monomer band was visible (lane 3). When p53 monomer was folded without tES, it failed to tetramerize (lane 7). In lane 5, 50 μM EDTA is added and p53 tetramers partially break into monomers and dimers (lane 5) (*n* = 3 independent experiments). [Note: CB—cage break] (**d**), tES–F116H(+/−) (**e**), tES–F116H(+) (**f**), tES–F116H(–), (**g**), p53 folded without tES. p53 folded with tES–F116H(+/−) shows a characteristic tetramer denaturation peak at 50 °C. Addition of mAb1620 results in a novel complex peak at ~95 °C. Differential scanning fluorimetry of mAb240, and p53 and **h**, tES–F116H(+/−) **i**, tES-F116H(+) **j**, tES–F116H(–) **k**, p53 folded without tES. p53 denaturation is affected by conA (**l**) but not a scrambled sequence (**m**). AUC analysis of tES–F116H(+/−)-folded tetramers demonstrates complex formation in the presence of ConA (**n**) but not in the presence of scr-conA (**o**).

*E*. *coli* expresses recombinant p53 as an insoluble inclusion body and in vitro folding typically results in low yields^53^. We tested the hypothesis that encapsulation of p53 monomers in tES would promote structure formation without aggregation and result in spontaneous tetramer formation upon release from the shell. A single monomer was encapsulated within tES–F116H(+) and tES–F116H(+/–), however, no encapsulation was observed with tES-F116H(–) (Fig. 6a). After protein release, a 174 kDa SEC peak was observed consistent with tetrameric p53 with the largest yield resulting from use of tES–F116H(+/–) (Fig. 6b). Prior to tES release, p53 was visualized using SDS-PAGE as a 47 kDa monomer. After tES release, p53 was predominantly seen as an ~180-kDa band that was attributed to the tetramer. Upon denaturation (boiling) of the same sample, the tetramer band disappeared and a 47-kDa monomer band was visible (Fig. 6c).

We found that p53 folded using tES–F116H(+/–) exhibits a clear dF/dt peak at a slightly higher denaturation temperature of 50 °C (Fig. 6d). Whereas PAb1620 antibody alone resulted in a negative dF/dt deflection at 78 °C, when PAb1620 was combined with p53, the 50 °C p53 denaturation peak disappeared, the PAb1620 negative deflection was reduced, and a new dF/dt peak at ~94 °C appeared, which was attributed to the p53:PAb1620 complex (Fig. 6d). A similar, but lesser, effect was seen with tES–F116H(+)-folded p53 (Fig. 6e), however, mixing PAb1620 with p53 folded without tES or p53 folded with tES–F116H(–) did not exhibit evidence of p53 stabilization or a PAb1620:p53 complex (Fig. 6f, g). Similar to PAb1620, PAb240 also exhibited a downward deflection of dF/dt at 78 °C. When mixed with tES–F116H(+/–)-folded p53, no evidence of complexation was seen. When PAb240 was mixed with p53 folded alone and with p53 folded using tES–F116H(–), a new denaturation peak (almost identical to the PAb1620:p53 complex peak) was observed at 94 °C, which was attributed to the PAb240:p53 complex (Fig. 6h–k).

p53 is a transcription factor and to test the function of tES–F116H-folded p53, we measured sequence-specific binding to consensus p53-response element (conA), a 20-b.p. DNA oligomer that binds tetrameric p53^54^. Similar to our observations with antibody stabilization, mixing conA with folded p53 ablated the 49 °C p53 denaturation peak in DSF in addition to a reduction of the DNA denaturation peak at 27 °C. Likewise, a new negative deflection peak centered around 55 °C was attributed to the conA:p53 complex. A scrambled version of conA showed no effect on p53 stabilization or complex formation (Fig. 6l). AUC analysis of tES–F116H(+/–)-folded p53 and conA further showed a 360-kDa complex consistent with a stable conA:p53 complex, whereas scrambled conA showed no evidence of complex formation (Fig. 6l, m). Overall, tES–F116H(+/–) provided optimal folding of p53. Although tES–F116H(+) also demonstrated positive effects on folding, tES–F116H(–) failed to produce p53 tetramers. tES–F116H(+/–) increased p53 crude soluble yield (950-fold), functional yield (10.5-fold), and specific activity (12-fold) (Figs. 1b, c; 6n, o).

### Comparisons of tES nanoparticle folding

Overall, we observed a consistent increase in soluble yields when POIs were folded with tES–F116H in comparison with POIs folded under identical conditions without tES (Fig. 1b, c). Although rFasxiator, rLuc and FFL exhibited a moderate increase (3–10-fold) in soluble yield, the majority of the POIs demonstrated a 50—>1000-fold increase in their soluble yields.

Consistent with our hypothesis of tES internalization of POI as a primary mechanism of POI folding, tES–F116H_subunits_:POI-encapsulation ratios for rFasxiator, rLuc, HSA, sAP, Omp2a, and p53 (Fig. 1d, supplementary Fig. 7) were found to be in accordance with the calculated POI molecular volumes (supplementary Fig. 2) and the steric upper limit of the tES–F116H shell interior. We modeled the internal volumes of tES charge variants^55^ using “1S3Q” as the template structure^56^ and all internal volumes were in accordance with the reported value of 324,900 Å^3^ for the native *Archaeoglobus fulgidus* ferritin [tES–F116H(+): 306,376 Å^3^; tES–F116H(+/–): 308,696 Å^3^, and tES–F116H(–): 303,928 Å^3^]^57^. In our attempt to characterize the encapsulation ratio of the two conotoxins (1.2–1.4 kDa), we found that the molecular weight of tES–F116H:POI dynamically decreased throughout the analytical ultracentrifugation (AUC) run (supplementary Fig. 7), leading us to hypothesize that the conotoxins are initially encapsulated but escape through the 4-nm triangular pores of tES during sedimentation.

tES shell assemblies are highly stable throughout the folding process. Specifically, all 12 tES–F116H:POI complexes demonstrated monodisperse, 12 nm radii in solution (supplementary Fig. 8) and the expected spherical morphology on transmission electron microscopy (supplementary Fig. 9). In addition, all three tES–F116H charge variants show stability of secondary structure to ~90 °C, and can undergo at least 10 rounds of pH titrated (pH 5.8–8.0) assembly and disassembly without precipitation or significant protein loss (Fig. 1g).

The relationship of tES–F116H:POI ratio to functional yields likewise demonstrated a sigmoidal distribution for all POI (Fig. 1d; supplementary Figure 2) and we did not observe any decrease in protein folding as a result of tES addition. The saturation point for tES–F116H_subunits_:POI functional yield generally followed the predicted steric limits of encapsulation, i.e., for tES_subunits_:conotoxins (60:150), tES_subunits_:rFasxiator/PLA_2_ (60:15), tES_subunits_:HRPc/GFPuv/rLuc (60:10), and tES_subunits_:FFL/HSA (60:5) (Fig. 1d, supplementary Figures 10–12). The encapsulation of POIs varied from 1 to 3 per shell as determined by AUC (Fig. 3). Further, the presence of empty shells was not observed at the optimal molar ratios for POI:tESF116H_subunits_. Taken as a whole, we conclude that the association of POI and tES occurs at high percentages (nearing 100%) of all tES shells formed. We also measured the percent recovery of POIs tested (~40–50%), with p53 being an outlier at 27% recovery (Fig. 1b, d).

Functional yields were further studied using differential scanning fluorimetry (DSF), a technique that monitors dye binding to exposed hydrophobic surfaces as a function of denaturing temperature^58^ (Fig. 1e). Each of three tES charge variants was tested under saturating conditions as determined in Fig. 1f. A consistent association between POI charge (individual zeta potentials reported in figure supplementary Fig. 10d), tES internal charge, and tES denaturation temperature can be seen (Fig. 1e) for all substrates, with opposite charge pairing providing thermal stabilization and the same charge pairing resulting in tES denaturation temperatures no different than tES alone (Fig. 1e, f). To interpret these results, we note that the mass of the tES shell (~500 kDa) is larger than any of the POI ( > 10-fold) thus, the thermal signal is likely to be dominated by the energetics of shell assembly and stabilization. For instance, positively charged POIs within tES–F116H(+) did not alter the denaturation temperature of 92.5 °C (DT_1_) for tES. However, when negatively charged POIs are encapsulated within tES-F116H(+), an upshift in the denaturation temperature (DT_2_ = 98 °C) was observed for tES, with ∆DT = 5.5 °C. Similar such observations were seen for the denaturation temperature of tES while encapsulating negatively charged or neutral POIs. We thus hypothesize that tES-specific interactions by POIs stabilize the tES assembly upon internalization^59^.

## Discussion

Nanoencapsulation within the 8-nm aqueous cavity of tES–F116H appears to exert a broad effect across protein classes and is governed by basic biophysical principles. The mechanism of this effect appears to recapitulate several qualities of the evolutionarily conserved chaperonin, GroEL/ES, which folds peptides in a similar-sized, 8-nm × 8-nm cylindrical cavity. Thermodynamic models suggest that the 8-nm scale of the GroEL/ES cavity plays a major role in the stabilization of proteins and studies, which empirically vary the volume of GroEL/ES demonstrate large effects on substrate folding^60,61^. Thus, the scale of the tES–F116H interior per se may play a significant role in our observed results. Because ~80% of translated monomeric proteins are less than 80 kDa, the majority of expressed monomers in nature would be potential candidates for tES^62^.

GroEL/ES is different from tES in that it uses ATP binding to alternate exposure of hydrophobic and hydrophilic residues and ATP hydrolysis to propagate a reaction cycle of iterative protein annealing^63^. Although the tES nanoparticles are not expected to undergo allosteric changes, the addition and removal of chaotropes while the POI remains confined inside the tES shell may approximate a single annealing step of GroEL/ES, albeit orders of magnitude slower. This is similar in theme to tES–F116H that are GroEL/ES variants, namely SR1/GroES, which lack ATPase ability yet retain protein-folding activity^64,65^.

Although we made an effort to study a diversity of POIs, there are also biases incorporated into our experiment. Primarily, we focused on proteins that have an in vitro activity assay, thus classes of proteins such as scaffolding and binding modulators are not included. Second, our sample set is based on proteins with monomeric molecular weight below 80 kDa (supplementary Fig. 2). Third, although all proteins are expressed and purified as denatured inclusion bodies, one of the proteins we attempted to study (trypsin) was unexpressable in *E. coli*, presumably due to intracellular toxicity. Thus, an additional important bias is that all studied proteins were biologically compatible with the expression host.

tES nanoparticles address critical gaps in the field of in vitro folding as they can be produced in large quantities, stored at room temperature, and exhibit structural stability in the harsh conditions often required of denatured protein solubilization^20^. tES may be helpful in further applications that require encapsulation within nanoscaled electrostatic environments^66,67^.

## Methods

### Materials

Inclusion bodies of rFasxiator, *α*- and *λ*-conotoxins were obtained from Prof. Kini Manjunatha’s lab (Dept. of biological sciences, NUS). The vectors used for cloning were the pBAD/HisB vector (Life Technologies) and pRSF1b expression vector (Merck). For transformation, chemically competent BL21 (DE3) *E. coli* cells (catalog #RH217-J40; Simply Science) and XL1 Blue *E. coli* cells (catalog #RH119-80; Simply Science) were used.

### Reagents

The reagents used were as follows: Kanamycin (ThermoFisher), Luria-Bertani (LB) agar (Axil Scientific), Omega bio-tek plasmid mini kit and gel extraction kit (Simply Science), Ampicillin (Axil Scientific), IPTG (Axil Scientific), L-arabinose (Sigma), Sodium Chloride (NaCl, Sigma), Tris (Sigma), Triton X-100 (Sigma), Hemin (Sigma), Calcium Chloride (CaCl_2_, Sigma), Magnesium Chloride (MgCl_2_, Sigma), L-glutathione oxidized (Sigma), β-mercaptoethanol (Sigma), Imidazole (Sigma), Phosphate-buffered saline (PBS, Sigma), Dithionite (Sigma), Sodium Thiosulfate (Sigma), Tween-20 (ThermoFisher), Ferrous Ammonium Sulfate (Sigma), Glycerol (Sigma), Potassium Iodide (Sigma), Coelentrazine substrate (Promega), chemiluminescent HRP substrate (Millipore), 3,3′,5,5′-tetramethylbenzidine (TMB) substrate (ThermoFisher), Sulfuric acid (H_2_SO_4_, Sigma), Ethylenediaminetetraacetic acid (EDTA, Sigma), Urea (1st Base), GuHCl (Sigma), Dithiothreitol (DTT, Sigma), and Thermo ScientificTM PierceTM c-Myc Tag IP/Co-IP Kit (cat. No. 23620), sPLA_2_ assay kit (Cayman), 5,5′-dithio-*bis*-(2-nitrobenzoic acid) (DTNB) (Sigma), specific Factor XIa (Merck), chromogenix S-2366 substrate (Diapharma), adenosine 5′-triphosphate (ATP) disodium salt (Sigma), D-luciferin potassium salt (Sigma), bovine serum albumin (BSA)(Santa Cruz Biotechnology), and 10X phosphate buffer saline (PBS)(Lonza). Antibodies used were as follows: mouse monoclonal anti-p53 antibody clone PAb421 (p53 Lab), mouse monoclonal anti-p53 (wild type) antibody clone PAb1620 (Sigma), mouse monoclonal anti-p53 antibody Pab240 (Abcam), and mouse monoclonal anti-hemagglutinin antibody (Sigma).

Antibodies used were as follows: concentration: mouse monoclonal anti-p53 antibody clone PAb421 (4 µM), mouse monoclonal anti-p53 (wild type) antibody clone PAb1620 (5 µM), and mouse monoclonal anti-p53 antibody Pab240 (5 µM), mouse monoclonal anti-hemagglutinin antibody (1 µg/ml).

### Inclusion body POI/tES–F116H nanoparticle preparation

pBAD/HisB and pRSF1b constructs containing genes for h6POI (GFPuv, rLuc, HRPc, HSA, FFL, and PLA_2_) (Genescript) and tES–F116H were transformed into chemically competent BL21(DE3) *E. coli* cells and grown on LB agar (Axil Scientific) plates with 50 µg/mL ampicillin and 25 µg/mL kanamycin, respectively, as reported in the previous study. For expression, a single positive colony was inoculated in 100 mL of LB broth with 100 µg/mL ampicillin and 50 µg/mL kanamycin selection. After overnight incubation at 37 °C, 12.5 mL of the starter culture was used to inoculate 500 mL of LB broth and was allowed to grow till absorbance (OD600) of 0.4 was reached. Protein expression was induced with 0.4 mM IPTG (pRSF vector) (Axil Scientific) and 0.1% L-arabinose (pBAD vector) (Sigma Aldrich) and incubated for 4 h at 37 °C. The cells were then centrifuged at 13,750 × *g* for 15 min.

For tES–F116H, the cell pellet was resuspended in lysis buffer (50 mM Tris-HCl, 150 mM NaCl, 0.1% Triton X-100, and 1 mM EDTA, pH 7.5), sonicated, and then centrifuged to separate the cell debris. The supernatant was subjected to two-step chromatography purification, which included hydrophobic interaction chromatography (HIC) using HiPrepTM Phenyl FF (low sub) 16/10 (GE Healthcare) followed by size-exclusion chromatography (SEC) using a Superdex S-200 10/300 GL column (GE Healthcare). The purity of SEC fraction was analyzed using SDS-PAGE. The buffers used were as follows: HIC buffer A: 25 mM Tris-HCl, 150 mM NaCl, and 1 M (NH_4_)_2_SO_4_, pH 7.5; HIC buffer B: 25 mM Tris-HCl, 150 mM NaCl, pH 7.5; SEC: 25 mM Tris-HCl, pH 8.0.

For h6POI, the cell pellet was resuspended in lysis buffer (25 mM Tris-HCl, 150 mM NaCl, 5 mM BME, and 1.5% Triton X-100, pH 7.5), sonicated, and centrifuged to separate the cell pellet. The cell pellet was then resuspended in washing buffers (25 mM Tris-HCl, 0.5% Triton X-100, 200 mM NaCl, 5 mM BME, pH 8.0, and 25 mM Tris-HCl, 0.5 M NaCl, 2 M urea, and 5 mM BME, pH 8.0) and centrifuged. The cell pellet was then resuspended in solubilization buffer, pH 8.0 (Table 1) to solubilize the inclusion bodies for 3–4 h (for FFL, solubilization time is 30 m), followed by centrifugation as reported previously. The supernatant was then subjected to Ni^2+^-NTA(Expedeon) chromatography purification, followed by the analysis of the eluted fraction using SDS-PAGE. Buffers used for Ni^2+^-NTA were as follows: Buffer A: Solubilization buffer, pH 8; Buffer B: Solubilization buffer, 500 mM imidazole, pH 8.

**Table 1:** Solubilization and refolding of proteins of interest (POIs).

Protein	Stabilization buffer	Refolding buffer		Calculated pI	tES charge	Diameter of tES encapsulated protein (nm)	
		Buffer	Additive			TEM	DLS
*α* conotoxin	S + 2 mM EDTA*	R_2_	NA	7.8	Negative	13.14 ± 1.01	11.78 ± 1.19
*λ* conotoxin	S + 2 mM EDTA*	R_2_	NA	8	Neutral	11.41 ± 1.53	11.55 ± 0.76
rFasxiator	S	R_1_	+ 1 mM GSSG/ 0.8 mM GSH/5 mM CaCl_2_	8.3	Negative	13.13 ± 1.23	11.73 ± 0.73
PLA_2_	S*	R_1_	+ 4 mM GSSG/2 mM cysteine/10 mM CaCl_2_	4.8	Positive	12.60 ± 1.39	12.27 ± 0.68
GFPuv	S*	R_1_	NA	5.8	Positive	13.10 ± 1.12	12.23 ± 0.28
HRPc	S*	R_1_	+ 0.35 mM GSSG/5 mM CaCl_2_/20 uM heme	6.3	Positive	13.16 ± 1,06	11.71 ± 0.86
rLuc	S*	R_1_	NA	5.86	Positive	12.87 ± 1.33	11.17 ± 0.79
FFL	S	R_1_	+ 50 mM KCl/ 3 mM MgCl_2_/1 mM DTT	6.69	Neutral	13.05 ± 1.23	11.53 ± 1.17
HSA	S	R_1_	+ 2 mM GSSG/1 mM GSH/1 mM EDTA	5.67	Positive	11. 99 ± 1.42	12.08 ± 1.24
sAP	S	R_1_	+ 0.8 mM GSSG/ 1 mM GSH/2 mM + MgCl_2_/4 mM ZnCl_2_	4.63	Positive	13.23 ± 1.15	NA
Omp2a	S	R_1_	+ 0.005% Triton-X	4.3	Positive	12.27 ± 2.06	NA
p53	S	R1	(a)2 mM DTT/ 1 mM ZnCl_2_/(b) 1 mMZncl2	6.33	Neutral	12.45 ± 1.46	NA

S: 50 mM Tris-Hcl - pH 8.0, 8 M Urea (or 6 M GuHcl), 0.1-0.2 M NaCl, 5 mM DTT (or 10 mM BME), *Heat at 60 °C for 30 min, R1: 50–100 mM Tris-Hcl - pH 8.0, 50-400 mM NaCl, 1–7% Glycerol, R2: 100 mM Tris-Hcl - pH 8.0, 2 mM EDTA, 1 mM GSSG,0.8 mM GSH, PLA_2_: Phospholipase A_2_, GFPuv: Green Fluorescent Protein, HRPc: Horseradish Peroxidase, rLuc: Renilla Luciferase, FFL: Firefly Luciferase, HSA: Human Serum Albumin, sAP: Shrimp Alkaline Phosphotase. Details regarding solubilization buffer, refolding buffer, pI of POI, tES charge, diameter of tES:POI assembly analysed by DLS and TEM.

pBAD/HisB constructs containing multimeric protein genes for h6POI (AP, Omp2a) (Genescript) and Pet22b plasmid (p53 lab) were transformed into chemically competent BL21(DE3) *E. coli* cells and grown on LB agar plates (Axil Scientific). For expression, a single positive colony was inoculated in 100 mL of LB broth with 100 µg/mL ampicillin and incubated overnight at 37 °C for h6POI and 30 °C for p53. Following the incubation, 12.5 mL of starter culture was used to inoculate 500 mL of LB broth and was allowed to grow till absorbance (OD600) of 0.4 was reached. Protein expression was induced by 0.1% L-arabinose (pBAD) and incubated for 4 h at 37 °C. For p53, expression was induced by 0.5 mM IPTG and 0.1 mM ZnCl_2_ and incubated at 30 °C for 5 h. The cells were then centrifuged at 13,750 × *g* for 15 min. For h6POI, the cell pellet was resuspended in lysis buffer (1.5% Triton X-100, 25 mM Tris-HCl, and 150 mM NaCl, pH 8.0), sonicated, and centrifuged to separate cell pellet. The cell pellet was then resuspended in washing buffers (0.5% Triton X-100, 25 mM Tris-HCl, 200 mM NaCl, pH 8.0, and 25 mM Tris-HCl, 0.5 M NaCl, 2 M urea, and 2 mM DTT, pH 8.0) and centrifuged. For p53, lysis and washing buffers were supplemented with 10 mM DTT. For Omp2a and AP, 10 mM β-ME was added to the lysis and washing buffers. The cell pellet was then resuspended in solubilization buffer, pH 8.0 (Table 1) to solubilize the inclusion bodies for 3–4 h, followed by centrifugation as reported previously. The supernatant was then subjected to Ni^2+^-NTA(Expedeon) chromatography purification, followed by the analysis of the eluted fraction using SDS-PAGE. Buffers used for Ni^2+^-NTA were as follows: Buffer A: Solubilization buffer, pH 8; Buffer B: Solubilization buffer, 500 mM imidazole, pH 8.

### In vitro folding

The purified tES–F116H variants were acidified for shell disassembly with 25 mM Tris-citrate buffer, pH 5.8, for 30 min followed by the isolation of the subunits using size-exclusion chromatography (SEC). The monomeric POIs purified from inclusion bodies were incubated with subunits (pH 5.8) at different ratios, until the optimum tES–F116H_subunits_:POI ratio was reached for maximum encapsulation efficiency. The pH of the mixture was then adjusted to 8.0 and the sample was incubated at room temperature for 30 min, followed by overnight dialysis of the mixture in folding buffer (Table 1) using Slide-A-Lyzer^®^ Dialysis Cassette G2 (Thermo Scientific). The folded protein encapsulated in tES was purified, the fractions acidified and subjected to a second SEC to separate the refolded proteins from the subunits. In the case of partial release of the POI from the tES, high-salt concentrations of 500 mM NaCl were used with release buffers (25 mM Tris, pH 5.8).

The refolded protein was purified using SEC. The SEC fractions were collected and checked for activity. The purification step with SEC also served as a buffer exchange to lower NaCl concentrations of 75–100 mM. The folded-protein fractions were then collected and analyzed for their functional activity. For the conotoxins, the functional fractions were analyzed using a well-characterized UPLC–QTOF mass spectrometry assay with positive standards. For the conotoxins, the pH of the mixture was adjusted to eight and incubated at room temperature for 30 min. The mixture was diluted slowly using stepwise addition of the refolding buffer, finally obtaining 10-fold dilution of the mixture. The diluted mixture was incubated overnight and then purified using SEC. The SEC fractions were collected and acidified for shell disassembly, following subjection to another SEC using 25 mM Tris-citrate, 0.5 M NaCl, pH 5.8, and buffer for separating refolded conotoxins from tES subunits. SEC elutes containing the conotoxins were analyzed using UPLC–QTOF system.

Optimal ratios of tES–F116H_subunits_:POI for functional yield were determined for nine monomeric POIs. POIs were titrated against a fixed concentration of tES and after removal of denaturant and protein aggregates, when possible, the soluble fraction was quantified using individual activity assays generally performed while the monomer was still inside the shell (conotoxin and rFasxiator assays required release from the shell).

Solubilized h6POI (Omp2a, AP) were incubated in the presence of acidified disassembled tES subunits (in 25 mM Tris-HCl, pH 5.8) in optimum tES–POI molar ratio of 60:5 for maximum encapsulation efficiency. For p53, the optimum tES–POI molar ratio was 60:10 (due to near neutral net surface charge of p53). The pH of the mixture was adjusted to 8 and the sample was incubated at room temperature for 30 min, followed by overnight dialysis of the mixture in refolding buffer (Table 1) using Slide-A-Lyzer^®^ Dialysis Cassette G2 (Thermo Scientific). For Omp2a, dialysis was done at 37 °C, while for p53 and AP, dialysis was done at 4 °C. Following dialysis, the pH of the mixture was changed to 5.75 to disassemble the exoshell and release of refolded monomer. For p53 and AP, after release, the protein was purified using Ni^2+^- NTA chromatography and then the protein was incubated at 4 °C for 60 min to facilitate multimerization. For Omp2a, following the Ni^2+^- NTA purification, the protein was incubated at 37 °C for nine days to facilitate the multimerization.

### Characterization of nanoparticles

The particle size and distribution of tES(+)F116H and tES–F116H/POI were determined by dynamic light scattering (DLS) (Nanobrook Omni, Brookhaven), using disposable cuvettes at 24 °C and the average hydrodynamic diameter was determined by taking an arithmetic average of 10 runs. The morphologies of the nanoparticles were analyzed using transmission electron microscopy (TEM, JEOL JEM-1220 TEM) and their zeta potentials were measured using Zeta-Plus analyzer (Nanobrook Omni, Brookhaven).

### In vitro assays

GFPuv and HSA activities were determined through fluorescence assays, HRPc and rFasxiator activities were determined through colorimetric assays, and rLuc and FFL activities were determined through luminescence assays, respectively, from purified proteins. All reactions were performed at least in triplicates. Bar graph represents quantification as mean ± SE. Fluorescence of GFPuv and HSA was read at 508 and 340 nm in a 96-well black polystyrene plate (Fisher Scientific) with excitation wavelength fixed at 395- and 282-nm wavelength, respectively. For HSA, tES exoshells were subjected to cage break and the protein was separated from the subunits by Ni^2+^- NTA chromatography. Following the purification, the fluorescence of refolded HSA was measured. The activity of HRPc was assayed using TMB substrate in a 96-well crystal-clear polystyrene plate (Greiner Bio-One). Purified fractions were incubated in an assay buffer containing 25 mM Tris-HCl, pH 8.0, 5 mM CaCl_2_, and 2.5 µM hemin for 5 min. TMB substrate was added for color development and the reaction stopped using 2 M H_2_SO_4_ after 5 min. Absorbance was recorded at 450 nm. rFasxiator selectively inhibits FXIa activity, thus inhibiting FXIa cleavage of Chromogenix S-2366 substrate (Diapharma). In all, 50 μl of rFasxiator was incubated with 50 μl of specific factor XIa (FXIa) (Merck) in 50 mM Tris, pH 5.8, 150 mM NaCl, and 5 mM CaCl_2_ at room temperature (24 °C) for 30 min. It was followed by the addition of 50 μl of Chromogenix S-2366 substrate (3 mM) and the cleavage of the substrate was recorded at 405 nm for 4 h. All rLuc and FFL reactions took place at ambient temperature (24–27 °C) in a 96-well plate with white interior. For rLuc, luciferase activity was evaluated using *Renilla* luciferase kit (Promega) with some modifications. The reaction was initiated by injecting 50 µL of *Renilla* luciferase assay reagent (1:1,000 dilution of coelenterazine in the assay buffer). For FFL, luciferase activity was evaluated using D-luciferin potassium salt as a substrate. The reaction was initiated by injecting 50 µl of buffer containing D-luciferin potassium salt (final concentration 200 µM), 50 mM Tris-Cl, pH 7.5, 10 mM MgCl_2_, 2 mM EDTA, 100 µM ATP, and 0.1% BSA, to a 50-µl protein sample. For both proteins, the assay reagents were protected from light at all times by covering the tubes with an aluminum foil. The signal was integrated for 1 min with a 2-s delay and was reported in RLU. All readings were recorded on a Perkin Elmer Plate reader. Appropriate controls were used in each case to minimize background. PLA_2_ activity was measured using sPLA_2_ assay kit (Cayman) in a 96-well crystal-clear polystyrene plate (Greiner Bio-One), which uses 1,2-dithio analog of diheptanoyl phosphatidylcholine as a substrate. PLA_2_ hydrolyzes thio-ester bond at the *sn*-2 position, which releases free thiols that are detected using 5,5′-dithio-*bis*-(2-nitrobenzoic acid) (DTNB). About 15 μl of refolded protein (in 25 mM Tris-HCl, 10 mM CaCl_2_, pH 8.0 buffer) was mixed with 10 μl of DTNB, and the reaction was initiated by adding 200 μl of substrate solution to the mixture. The mixture was then incubated for 2 h, after which the absorbance was recorded at 405 nm.

### Thermal stability measurement

Thermal stability of the nanoparticles was determined using a protein thermal shift assay kit (Thermofisher) in 384-well PCR plates. About 20 μl of reaction mixture containing 12.5 μl of sample, 2.5 μl of Protein thermal shift dye (8X), and 5 μl of Protein thermal shift buffer was dispensed in each well. The PCR plates were then sealed with optical seal and centrifuged to remove the air bubbles in the mixture. Thermal scanning (25–99.9 °C at 0.05 °C/s) was done using real-time PCR system (QuantStudio 7 Flex System, Applied Biosystems) and the fluorescence intensity was measured every 10 s. Analysis of the denaturation curve and determination of denaturation temperature was done using QuantStudio Real-Time PCR software (Applied Biosystems). All reactions were performed at least in triplicates. The curve represents quantification as mean ± SE.

### Encapsulation-efficiency measurement

Experimentally, a Beckman Coulter ProteomeLabTM XL-I was used to determine the encapsulation efficiency of tES–F116H for different POIs. The tES–F116H/POI samples (1 µM) in PBS buffer and reference PBS buffer were loaded into the two holes of an Epon cell with sapphire windows. These cells were housed in a four-hole rotor (Ti-60, Beckman-Coulter), with one hole occupied by a reference cell for radial calibration and kept at a constant temperature of 20 °C. The optical densities of the samples at 280 nm were measured after every 7 min as a function of time and cell radius for tracking the sedimentation of the tES–F116H with and without POI encapsulation, till all the particles had sedimented to the bottom of the cells. The experiment was continued at a constant rotation speed of 40,000 × *g* for 7.5 h. The data were analyzed with SEDFIT and SEDNTERP software. The quality of the fit for the AUC curve was assessed using residuals plots, rmsd (<0.1), and *Z*-value (<15) (Tables 2, 3)^148^.

**Table 2:** rmsd and runs test *Z* values of AUC runs.

Protein	rmsd	Runs test *Z*
tES(+)F116H	0.005061	1.06
tES(+)F116H:HSA (60:1)	0.006266	3.39
tES(+)F116H:HSA (60:3)	0.006247	12
tES(+)F116H:HSA (60:5)	0.00533	6.85
tES(+)F116H:rLuc (60:1)	0.006272	2.13
tES(+)F116H:rLuc (60:3)	0.004917	4.72
tES(+)F116H:rLuc (60:5)	0.007101	0.59
tES(+)F116H:rLuc (60:8)	0.00634	0.56
tES(+)F116H:rLuc (60:10)	0.006580	2.39
tES(−)F116H:rFasxiator (60:1)	0.004283	1.64
tES(−)F116H:rFasxiator (60:3)	0.004570	0.31
tES(−)F116H:rFasxiator (60:5)	0.00592	3.59
tES(−)F116H:rFasxiator (60:8)	0.004917	8.67
tES(−)F116H:rFasxiator (60:10)	0.004005	5.02
tES(−)F116H:rFasxiator (60:12)	0.004448	0.48
tES(−)F116H:rFasxiator (60:15)	0.00415	7.19

Determining the quality of fit for AUC curve for different molar ratios of tES to HSA, rLuc and rFasxiator respectively.

**Table 3:** rmsd and runs test *Z* values of AUC runs.

Protein	rmsd	Runs test Z
tES(+)F116H:HSA	0.005330	6.85
tES(+/–)F116H:HSA	0.006843	9.50
tES(–)F116H:HSA	0.006145	2.53
tES(+)F116H:rLuc	0.006580	2.39
tES(+/–)F116H:rLuc	0.007429	9.07
tES(–)F116H:rLuc	0.005081	4.59
tES(+)F116H:rFasxiator	0.003675	5.92
tES(+/–)F116H:rFasxiator	0.003491	1.29
tES(–)F116H:rFasxiator	0.006904	4.58

Determining the quality of fit for AUC curve for HSA, rLuc and rFasxiator encapsulated within tES(+)F116H, tES(+/–)F116H and tES(–)F116H respectively.

### Release of functional protein monomers and specific activity measurement

Engineered pH-responsive tES–F116H shells containing the POI—HSA, FFL, rFasxiator, PLA_2_, GFPuv, HRPc, and rLuc—were subjected to cage break in 50 mM Tris-citrate, 500 mM NaCl, pH 5.8, as described above. Following the cage break, the sample was subjected to SEC and the POI activity was measured in SEC fractions. The POI was then separated from tES–F116H subunits in the eluted fractions using Ni^2+^-NTA chromatography as described above. The concentration of POI was measured in using Nanodrop (DeNovix) and POI activity was determined as described. Using POI activity and concentration values, the specific activity was calculated. tES–F116H shells containing *α-* and *λ*-conotoxins were subjected to cage break in 25 mM Tris-citrate, 0.5 M NaCl, pH 5.8, and the eluted fractions were analyzed using UPLC–QTOF systems. All reactions were performed at least in triplicates. Bar graph represents quantification as mean ± SE.

### UPLC–QTOF analysis

Concentrated and pooled HPLC fractions were run on UPLC–QTOF systems (1290 Infinity II LC + Agilent 6550B QTOF). Chromatographic separation was achieved with a linear gradient using mobile phase A (0.1% formic acid in water) and B (0.2% formic acid in acetonitrile) at a flow rate of 0.40 mL/min on a Phenomenex Kinetex 2.6-μm XB-C18 100 Å 100 × 2.1 mm column with a total run time of 10 min. Electrospray-positive ionization (ESI+) mode was selected, and the mass/charge was acquired in the 100–1700 m/z range.

The chromatogram was obtained by extracting the ion count of the dominant m/z species of conotoxin (tolerating an error of 50 ppm) against acquisition time. The retention time is then compared against synthetic peptide standards to determine conformation and relative quantity of the conotoxins.

### In vitro assays for multimeric POIs

Fluorescence of refolded AP was read at 340 nm in a 96-well black polystyrene plate (Fisher Scientific) with excitation wavelength fixed at 282-nm wavelength. Omp2a fluorescence was read at 351 nm with excitation wavelength fixed at 286 nm. AP activity was measured using alkaline phosphatase assay kit (Abcam) in a 96-well crystal-clear polystyrene plate (Greiner Bio-One), which uses p-nitrophenyl phosphate (pNPP) as substrate. pNPP turns yellow when dephosphorylated by AP. About 80 μl of refolded protein (1 μM in 25 mM Tris-HCl, 2 mM MgCl_2_, and 4 mM ZnCl_2_, pH 8.0 buffer) was mixed with 50 μl of 5 mM pNPP solution, and the plate was incubated at 25 °C for 120 min in the dark. The reaction was stopped by adding 20 μl of stop solution and the absorbance was recorded at 405 nm. For Omp2a, 0.5 ml of sample was taken after one day, three days, six days, and nine days, respectively and was subjected to size-exclusion chromatography. The eluted peak was then compared with gel-filtration markers to determine the formation of Omp2a trimer. About 12% SDS gel was used to analyze the purified multimerized p53 and Omp2a. All reactions were performed at least in triplicates. Bar graph represents quantification as mean ± SE.

### Encapsulation-efficiency measurement

Experimentally, a Beckman Coulter ProteomeLabTM XL-I was used to determine the encapsulation efficiency of tES–F116H for different POIs as well as the multimerization of POIs. The tES–F116H:POI samples (1 µM) and POI (0.5 mg/mL) in refolding buffers and reference refolding buffers were loaded into the two holes of an Epon cell with sapphire windows. These cells were housed in a four-hole rotor (Ti-60, Beckman–Coulter), with one hole occupied by a reference cell for radial calibration and kept at a constant temperature of 20 °C. The optical densities of the samples at 280 nm were measured after every 7 min as a function of time and cell radius for tracking the sedimentation, till all the particles had sedimented to the bottom of the cells. The experiment was continued at a constant rotation speed of 40,000 × *g* for 7.5 h. AUC experiments to analyze p53-binding properties were done at a constant rotation speed of 10,000 × *g* for 7.5 h. The data were analyzed with SEDFIT and SEDNTERP software. The quality of the fit for the AUC curve was assessed using rmsd (<0.1) and *Z*-value (<15) (Tables 4–6)^148^.

**Table 4:** rmsd and runs test Z values of AUC runs (AP).

Protein	rmsd	Runs test Z
tES(+)F116H: AP (0 mM Mg, 4 mM Zn) cb	0.007639	11.57
tES(+)F116H: AP (1 mM Mg, 4 mM Zn) cb	0.007541	12.24
tES(+)F116H: AP (2 mM Mg, 4 mM Zn) cb	0.008539	11.07
tES(+)F116H: AP (4 mM Mg, 4 mM Zn) cb	0.00533	10
tES(+)F116H: AP (2 mM Mg, 0 mM Zn) cb	0.006777	14.35
tES(+)F116H: AP (2 mM Mg, 2 mM Zn) cb	0.007991	12
tES(+)F116H: AP (2 mM Mg, 4 mM Zn) cb	0.008152	11.01
tES(+)F116H: AP (2 mM Mg, 8 mM Zn) cb	0.009427	4.56
tES(+/–)F116H: AP (2 mM Mg, 4 mM Zn)	0.008188	9.04
tES(–)F116H: AP (2 mM Mg, 4 mM Zn)	0.008328	2.74
tES(+)F116H: AP (2 mM Mg, 4 mM Zn)	0.008280	7.37
tES(+/–)F116H: AP cb (2 mM Mg, 4 mM Zn)	0.009111	4.40
tES(–)F116H: AP cb (2 mM Mg, 4 mM Zn)	0.009477	7.30
Refolded AP (2 mM Mg, 4 mM Zn)	0.009967	3.08

Determining the quality of fit for AUC curve for AP encapsulated, refolded and released from tES(+)F116H under different concentrations of cofactors Zn^2+^ and Mg^2+^, tES(+/–)F116H and tES(–)F116H and AP refolded without tES.

**Table 5:** rmsd and runs test *Z* values of AUC runs (Omp2a).

Protein	rmsd	Runs test Z
tES(+)F116H: Omp2a cb (1 day)	0.0079511	5.77
tES(+)F116H: Omp2a cb (3 days)	0.006889	6.60
tES(+)F116H: Omp2a cb (6 days)	0.005686	15.38
tES(+)F116H: Omp2a cb (9 days)	0.007067	0.73
tES(+)F116H: Omp2a (9 days)	0.009519	1.71
tES(+/–)F116H: Omp2a (9 days)	0.009693	0.95
tES(–)F116H: Omp2a (9 days)	0.008411	12.18
tES(+/–)F116H: Omp2a cb (9 days)	0.007434	9.16
tES(–)F116H: Omp2a cb (9 days)	0.007360	12.70
Refolded Omp2a (9 days)	0.010069	15.59

Determining the quality of fit for AUC curve for Omp2a encapsulated, refolded and released from tES(+)F116H incubated for 1, 3, 6 and 9 days at 37 °C, tES(+/–)F116H and tES(-)F116H and Omp2a refolded without tES, incubated at 37 °C for nine days.

**Table 6:** rmsd and runs test *Z* values of AUC runs (p53).

Protein	rmsd	Runs test Z
PAb1620	0.00888	1.34
PAb1620 + p53 (tES(+/–)F116H)	0.007712	0.63
PAb1620 + refoldedp53	0.006290	2.39
PAb240	0.009902	8.09
PAb240 + p53 (tES(+/–)F116H)	0.009038	0.98
PAb240 + refoldedp53	0.006242	2.65
conA + p53 (tES(+/–)F116H)	0.007855	1.17
p53 (tES(+/–)F116H)	0.006754	9.70
conA	0.008237	10.83
Scr-conA + p53 (tES(+/–)F116H)	0.007977	12.92
Scr-conA	0.009294	8.26
tES(+/–)F116H:p53	0.009285	3.15
tES(+)F116H:p53	0.007743	4
tES(–)F116H:p53	0.006258	5.61
p53(tES(+)F116H)	0.007469	3.23
p53(tES(–)F116H)	0.008188	6.05
Refoldedp53	0.007261	0.51

Determining the quality of fit for AUC curve for p53 encapsulated, refolded and released from tES(+)F116H, tES(+/–)F116H and tES(–)F116H; p53-DNA mixture (p53 refolded from tES(+/–)F116H); p53 antibody complex (PAb1620 and PAb240) (p53 refolded using tES(+/–)F116H and without tES).

### p53-binding assays

To test the folding efficiency of p53, PAb240 and PAb1620 were used. PAb1620 specifically recognizes p53 in its wild-type conformation, i.e., the conformation of the protein when it is correctly folded, by binding to the residues Arg156, Leu206, Arg209, and Gln/Asn210^188^. PAb240 interacts with the epitope that is structurally hidden in the core of wild-type p53 (amino acids 213–217), but is exposed if the protein is denatured or misfolded^189^. About 100 μl of antibody (5 μM) is incubated with 100 μl of purified refolded p53 (5 μM) at 4 °C on a rotor for 1.5 h to facilitate antibody binding. After incubation, the p53-antibody complex was analyzed using analytical ultracentrifuge and differential scanning fluorimetry.

Binding efficiency of p53 was analyzed using consensus p53-response element (conA) and scr-conA (jumbled conA sequence) primers (Table 7). The conA has a high binding affinity to p53, while scr-conA binds weakly or not at all to the p53 tetramer. About 100 μl of purified p53 (4 μM) was incubated with 100 μl of primers (4 μM) and 50 μl of PAb421 antibody (4 μM) at 4 °C on a rotor for 1.5 h to facilitate p53–DNA-complex formation. After incubation, the complex was analyzed using differential scanning fluorimetry and analytical ultracentrifuge.

**Table 7:** Oligonucleotides.

Con A-TOP	5′ - GTT AGA GGG GCA TGT CCG GGC ATG TCC GGG CAG A - 3′
Con A-BOT	5′ - TCT GCC CGG ACA TGC CCG GAC ATG CCC CTC TAA C - 3′
Scr-con A-TOP	5′ - ACG GAG CGT GGC TCC GAG TAA GGC GGT AGC GTT G - 3′
Scr-conA-BOT	5′ - CAA CGC TAC CGC CTT ACT CGG AGC CAC GCT CCG T – 3′

### Oligonucleotides used

Specific activities for tES-folded p53 and p53 folded alone were calculated using area under the curve for the p53 ConA complex during analytical ultracentrifugation. As no complex formation was observed using p53 folded in isolation, this ratio was qualitatively described as >100-fold.

### Porin-channel measurement for Omp2a

Lipid-bilayer measurements were done by Prof. Mathias Winterhalter’s lab (Jacob’s University, Germany). Channel-recording apparatus consisted of a two-compartment Teflon chamber (~2.5 mL each) separated by a 25-μm-thick Teflon partition with an aperture of diameter ~100 μm for membrane formation (Supplementary Fig. 3). Bilayer lipid membranes were formed from 1,2-diphytanoyl-sn-glycero-phosphatidyl-choline (DPhPC) using the monolayer-opposition technique^190^. The aperture was pretreated with a hexadecane/hexane solution (1 % v/v) and allowed to cure for ~20 min to achieve solvent evaporation. The *trans*- and *cis*-sides of the chambers were filled with buffer solution, 1 M KCl, and 10 mM HEPES at pH 7.0, 10 μL of lipid in pentane (5 mg/ml) was added to both sides of the chamber, and the bilayer is formed after evaporation of pentane. Channel reconstitution is achieved by the addition of Omp2a into the cis-side of the chamber at a volume of 0.5 μL from Omp2a stock solutions (1 mg/ml) diluted ∼10^2^–10^5^ times by a solution of Genapol (1.1% v/v). Channel current traces were recorded with Ag/AgCl electrodes in agarose salt bridges containing 3 M KCl, or calomel electrodes containing a salt bridge (Metrohm AG) in the case of reversal-potential measurements. The cis-side of the chamber was considered the virtual ground, and measurements were done using the Axopatch 200B (Molecular Devices, LLC) patch-clamp amplifier in V-clamp mode (whole-cell *β* = 1) with a CV-203BU headstage. The output signal was filtered by a low-pass Bessel filter at 10 kHz, and saved at a sampling frequency of 50 kHz using an Axon Digidata 1440 A digitizer (Molecular Devices, LLC). Data analysis is performed with Clampfit 11.0.3 (Molecular Devices, LLC), Origin Lab 2018 (Northampton, MA, USA), and self-developed LabVIEW (National Instruments) data acquisition and analysis package for acquisition and analysis of reversal-potential measurements. Both single- and multichannel-insertion analysis was done for tES–Omp2a activity. Channel insertions achieved at 20 mV and/or 50 mV. Single-channel conductance was obtained by analyzing multichannel-insertion steps ΔI/V_m = G. Multichannel insertions of tES–Omp2a were obtained at V_m = + 50 mV (positive-membrane potential) and –50mV (negative-membrane potential). Insertion frequencies of ion channels into lipid bilayers do not reflect the functional concentration of ion channels within a solution. Thus, we defined the “active” form of Omp2a as the trimeric assembly molecular weight as observed in SEC. As no trimeric formation was observed using Omp2a folded in isolation, this ratio was qualitatively described as >100-fold.

### Reporting summary

Further information on research design is available in the Nature Research Reporting Summary linked to this article.

## Supplementary information

Supplementary Information

Reporting Summary

## Data Availability

All data required to reproduce this work are in the main manuscript and Supplementary material, or are available from the authors upon reasonable request. A Source Data file is available. Source data are provided with this paper.

## Source data

Source Data
