# Supplementary material for: A general approach to protein folding using thermostable exoshells
Source: Nat Commun. 2021 Sep 29;12:5720. doi: 10.1038/s41467-021-25996-4 (PMC8481291; doi:10.1038/s41467-021-25996-4)
Supplement: Supplementary file 1 — Supplementary Information [file 41467_2021_25996_MOESM1_ESM.pdf]

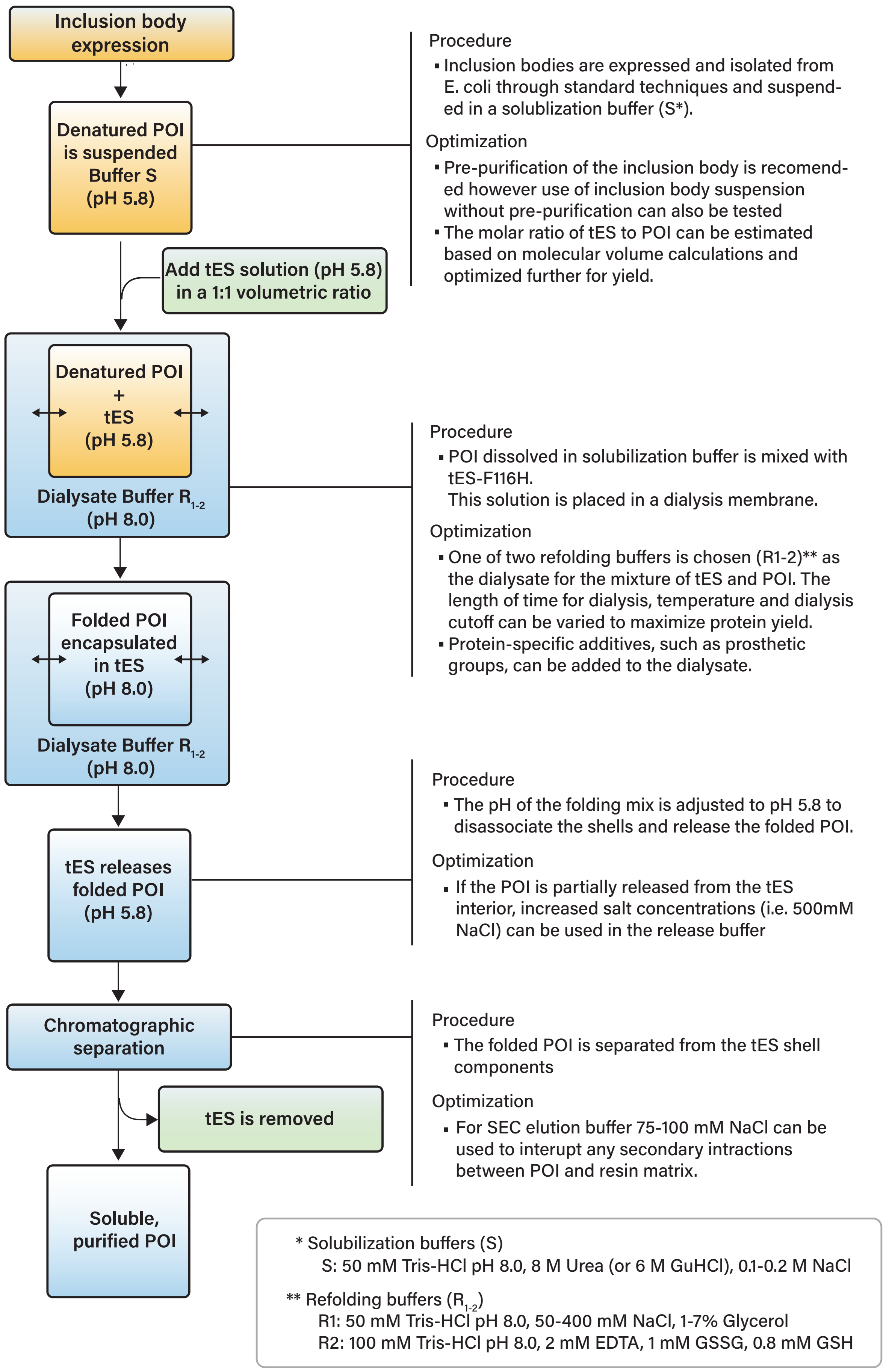

Supplementary Fig. 1| | A template protocol for protein folding. The selection of tES-F116H(+) vs tES-F116H(+/-) vs tES-F116H(-) is initially guided by charge complementation of the aggregate charge of the POI at neutral pH.

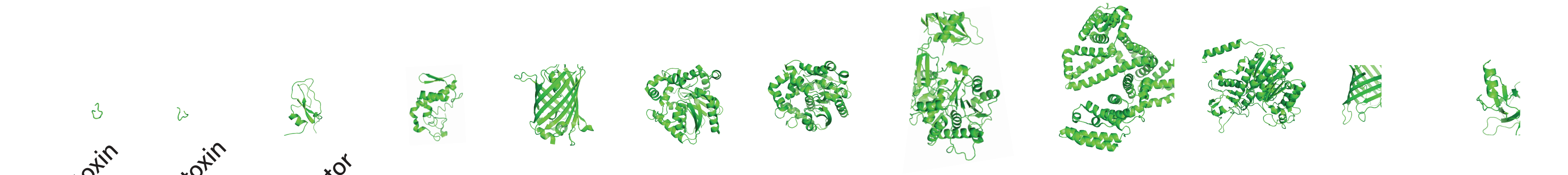

	α conotoxin	λ conotoxin	rFaxiator	PLA2	GFP	HRPc	rLuc	FFL	HSA	AP	Omp2a	p53
	1.35 kDa	1.24 kDa	12 kDa	17 kDa	27 kDa	34 kDa	36 kDa	60 kDa	66 kDa	52 kDa	39 kDa	43.7 KDa
PI of protein	7.8	8	8.3	4.8	5.80	6.30	5.86	6.69	5.67	4.63	4.3	6.33
Molucular Volume	2437 Å^3	1912 Å^3	16859 Å^3	12425 Å^3	23457 Å^3	30791 Å^3	32627 Å^3	55137 Å^3	59554 Å^3	47582 Å^3	61872 Å3	41484 Å^3
Charge at pH 7.0	0.8	1	3.5	-5.5	-6.2	-1.2	-13.4	-1	-15.7	-38.6	-22.6	-3.3
Disulfide (#)	2	2	3	7	0	4	0	0	17	2	0	4
Optimal tES	tES(-)	tES(+/-)	tES(-)	tES(+)	tES(+)	tES(+)	tES(+)	tES(+/-)	tES(+)	tES(+)	tES(+)	tES(+,+/-)
tES:POI encapsulation (max ratio)	N/A	N/A	1:3	N/A	N/A	N/A	1:2	N/A	1:1	1:1 monomer	1:1 monomer	1:1 monomer
tES:POI (max stabilization)	60:150	60:150	60:15	60:15	60:10	60:10	60:10	60:5	60:5	60:5	60:5	60:10
tES:POI (max functional yeild)	1:60	1:60	1:6	1:6	1:4	1:4	1:4	1:8	1:8	1:8	1:8	1:4
POI multimer	No	No	No	No	No	No	No	No	No	Yes	Yes	Yes
Source	Molluscs	Molluscs	Reptiles	Reptiles	Cnidarians	Plants	Cnidarians	Arthropods	Human	Arthropods	Bacteria	Human
Secondary Structure(%)	α helix : 41.7	N.A	α helix : 12.3<br>β strand : 24.6	α helix : 45.2<br>β strand : 8.1	α helix : 2.2<br>β strand : 50.7	α helix : 48<br>β strand : 2	α helix : 38.6<br>β strand : 18	α helix : 30.3<br>β strand : 21.3	α helix : 70<br>β strand : 0	α helix : 30.7<br>β strand : 18.3	α helix : 4.2<br>β strand : 71.3	α helix : 16.5<br>β strand : 21
Cysteine Density (%)	33	40	9	10	0.8	2.5	1	0.9	6	0.8	0.6	2.5

Supplementary Fig. 2| Comparative characteristics of monomeric and multimeric POIs. The molecular volumes of all the POIs were calculated using Voss Volume Voxelator program (Voss and Gerstein, Nucleic Acids Res. 2010). The PDB codes used were 1G2G (α conotoxin), 2B5P (λ conotoxin), 1JC6 (rFasxiator), 1PSJ (PLA2), 1H6R (GFP), 1GWU (HRPc), 2PSJ (rLuc), 5DV9 (FFL), 1AO6 (HSA) and 1K7H (sAP). For omp2a and p53, the monomeric structures were modelled using I-Tasser for molecular volume and secondary structure determination. The percent secondary structures for all other POIs were calculated using STRIDE (Heinig and Frishman, Nucleic Acids Res. 2004)

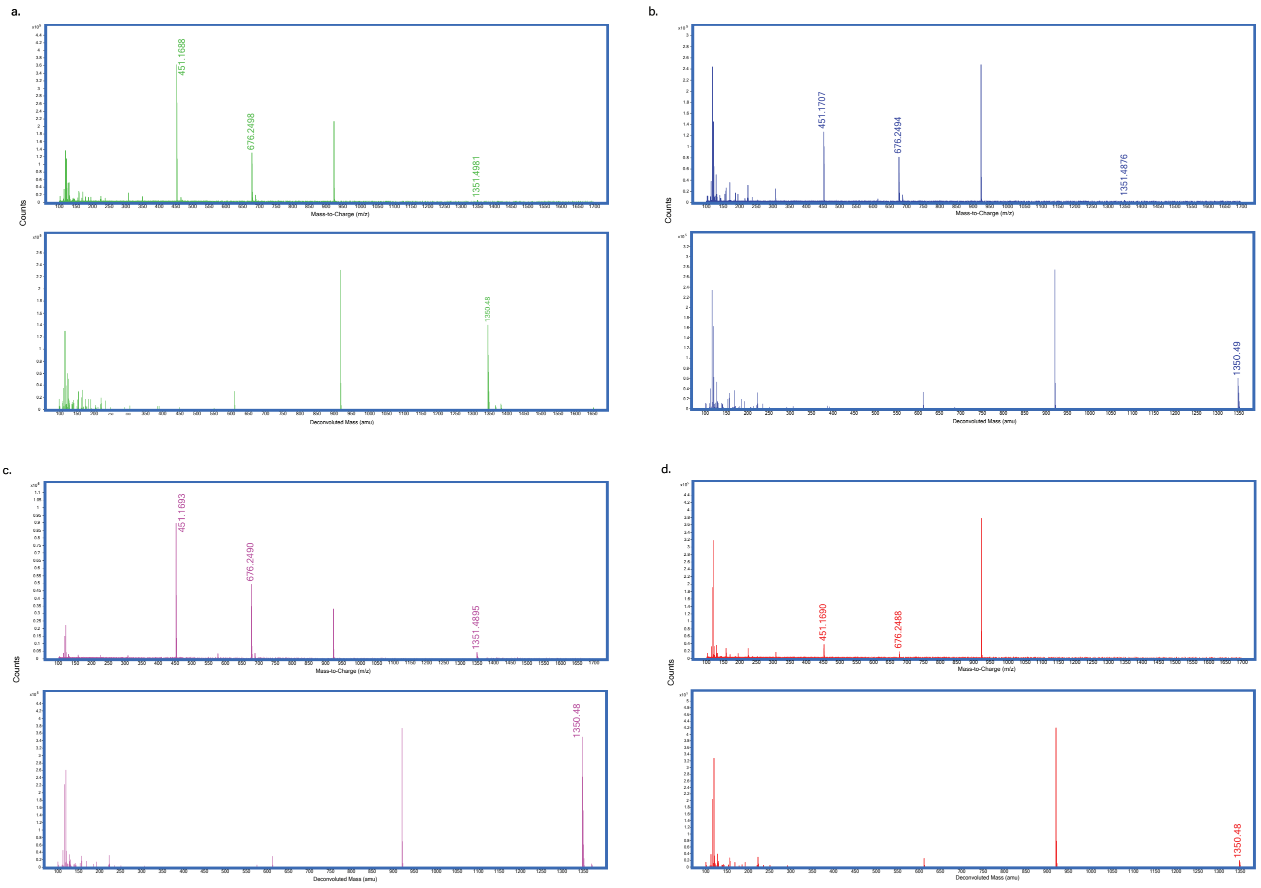

Supplementary Fig. 3 | Mass spectrometry profiles of globular conformation of α conotoxin folded in the presence and absence of tES: Raw mass/charge spectrum (m/z) (upper panel) and deconvoluted mass spectrum showing observed molecular masses (lower panel) of α conotoxin folded in vitro in presence of (a) tES(+)F116H, (b) tES(+/-)F116H and (c) tES(-)F116H and in (d) absence of tES were determined by QTOF. The protein is predominantly +3 charged, appearing with m/z of 451 amu. Protein has observed molecular mass of 1350.48 amu which closely matches with the calculated molecular mass of 1350.92 amu. In all cases, molecular weight of the oxidized α conotoxin shows a reduction of four mass units, reflective of the formation of the two disulfide bridges. m/z at 121 and 922 were from mass calibration standards.

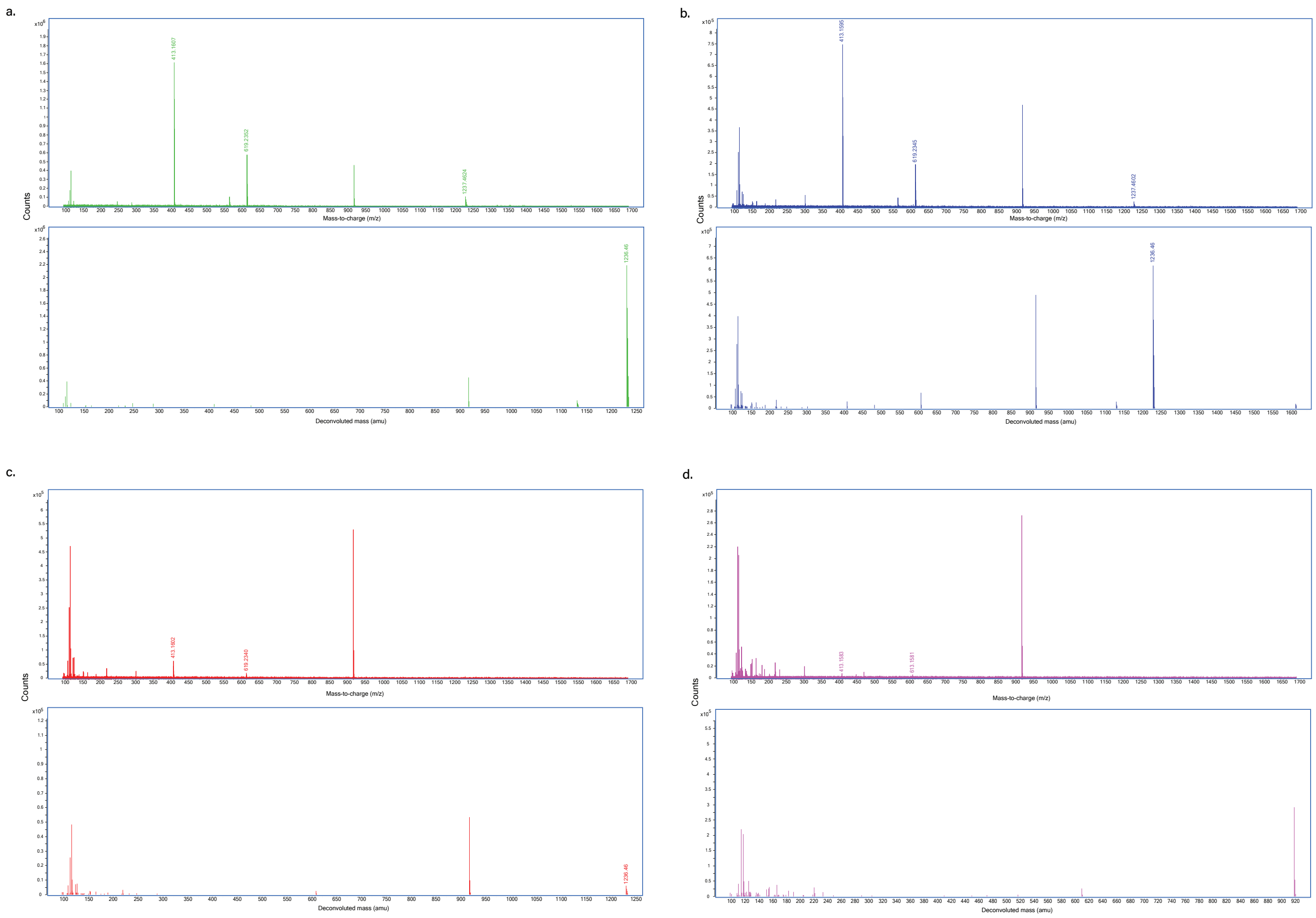

Supplementary Fig. 4 | Mass spectrometry profiles of ribbon conformation of λ conotoxin folded in presence and absence of tES: Raw mass/charge spectrum (m/z) (upper panel) and deconvoluted mass spectrum showing observed molecular masses (lower panel) of λ conotoxin folded in-vitro in presence of (a) tES-F116H(+), (b) tES-F116H(+/-) and (c) tES-F116H(-) and in (d) absence of tES were determined by QTOF. The protein is predominantly +3 charged, appearing with m/z of 413 amu. Protein has observed molecular mass of 1236.46 amu which closely matches with the calculated molecular mass of 1236.90 amu. In all cases, molecular weight of the oxidized λ conotoxin shows a reduction of four mass units, reflective of the formation of the two disulfide bridges. m/z at 121 and 922 were from mass calibration standards.

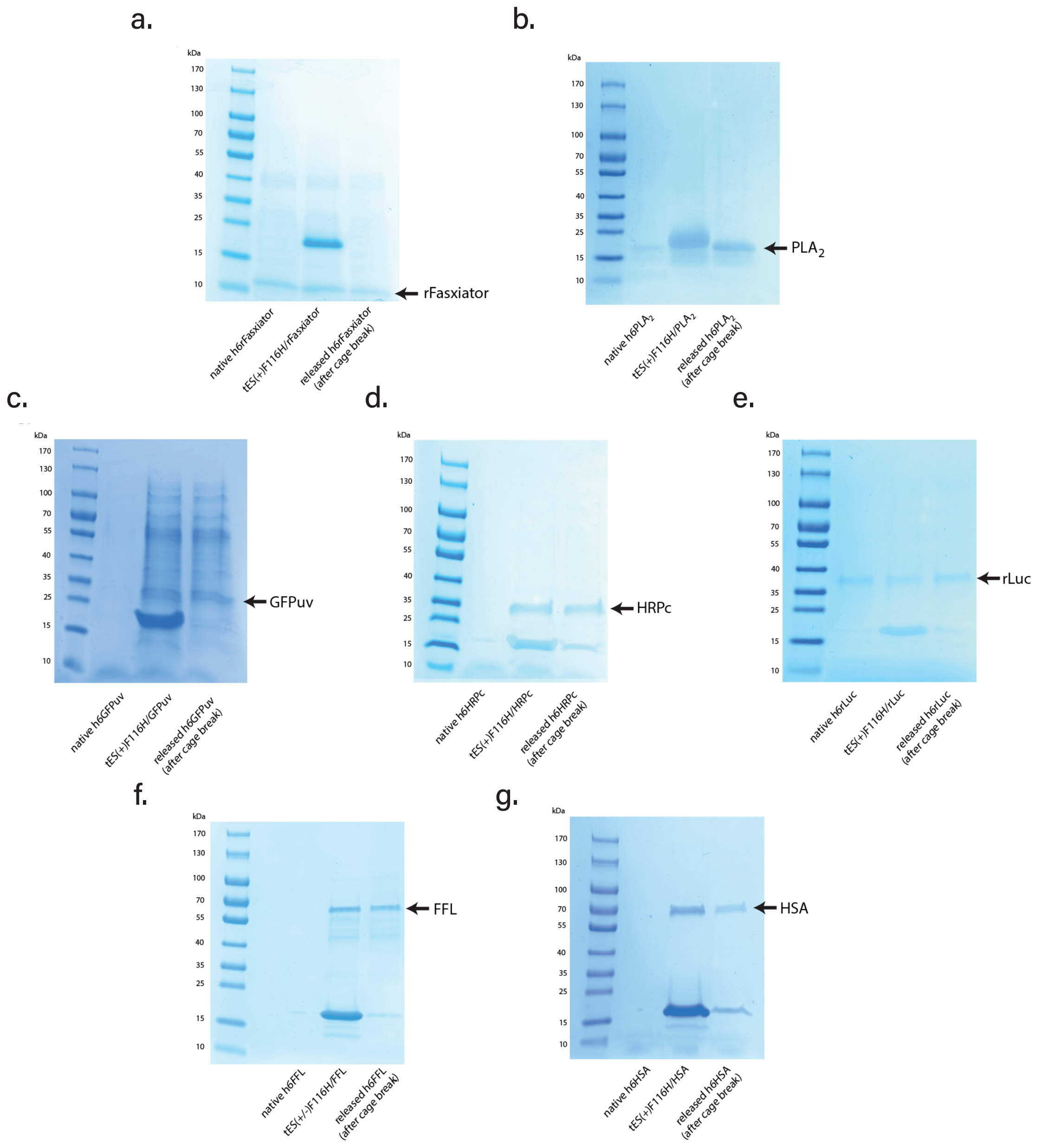

Supplementary Fig. 5 | Determining yield of in vitro folded POI in presence and absence of shells: SDS-PAGE gel of in vitro folded POI in absence of tES, in vitro folded POI in presence of tES and POI released and purified from tES after in vitro folding for (a) rFasxiator, (b) PLA_2, (c) GFPuv, (d) HRPc, (e) rLuc, (f) FFL and (g) HSA (n=3 independent experiments).

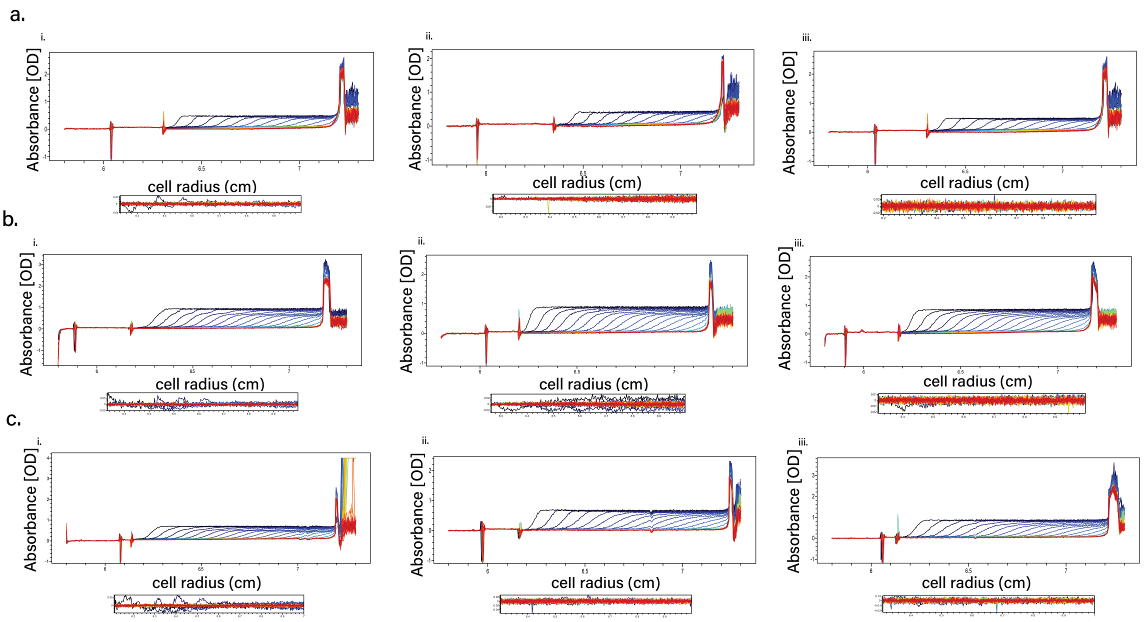

Supplementary Fig. 6 | Analysis of encapsulation with tES-F116H variants using Analytical Ultracentrifuge (AUC): Absorbance profiles (upper panel) and residual plots (lower panel) of (a) rFasxiator, (b) rLuc and (c) HSA encapsulated within (i) tES-F116H(+), (ii) tES-F116H(+/-) and (iii) tES-F116H(-) respectively at optimal molar ratios registered at 280 nm , in time intervals of 7 min for total experimental time of 7 h approximately.

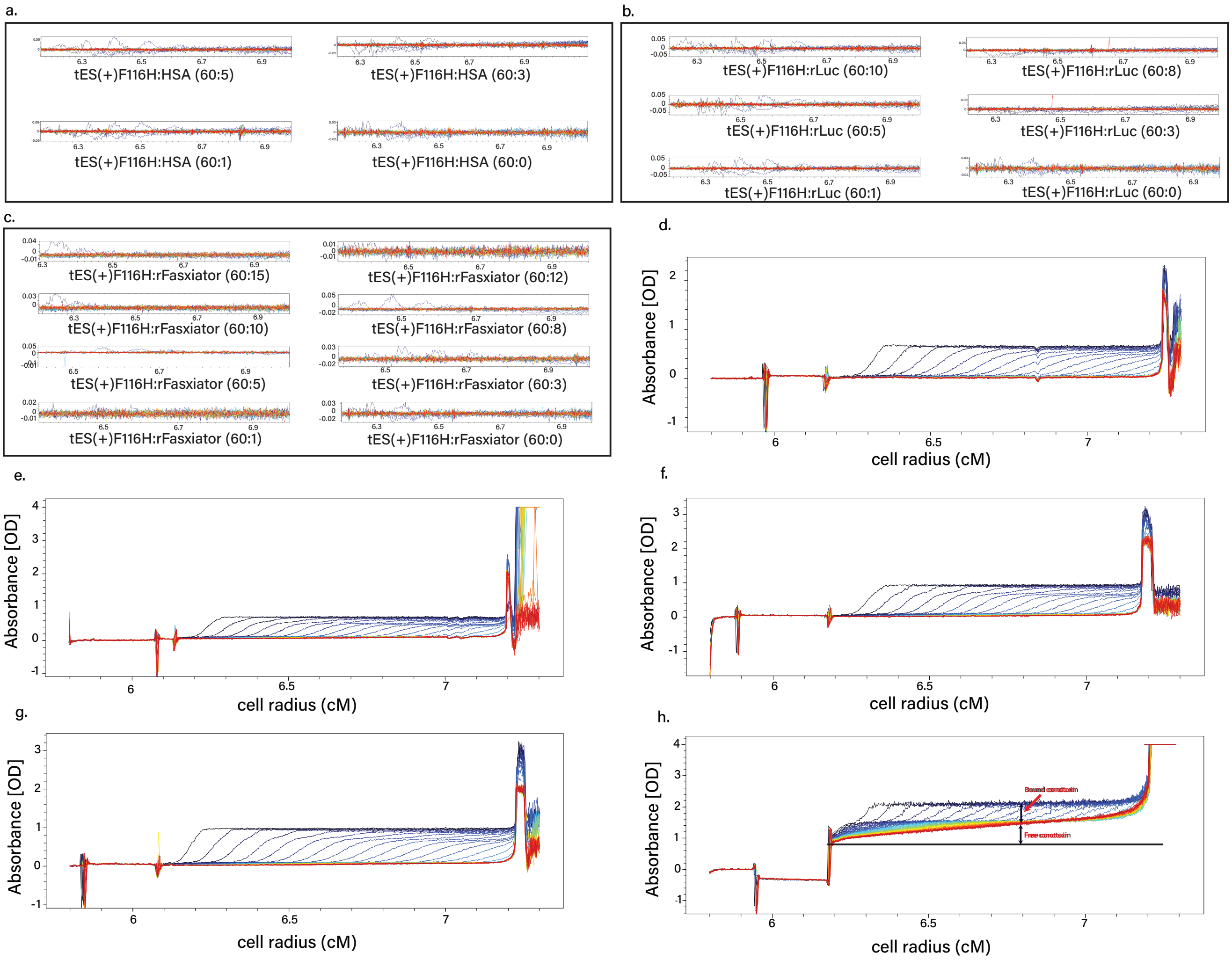

Supplementary Fig. 7| Analysis of encapsulation with increasing POI concentration using Analytical Ultracentrifuge (AUC): Residual plots (a,b,c) and absorbance profiles of (d) tES-F116H(+), (e) ttES-F116H(+):HSA, (f) tES-F116H(+):rLuc, (g) tES-F116H(-):rFasxiator, (h) tES-F116H(+):λ conotoxin at optimal molar ratios registered at 280 nm , in time intervals of 7 min for total experimental time of approximately 7 h. AUC profile for λ conotoxin shows decreasing sedimentation of tES encapsulated conotoxin and increasing presence of free conotoxin as the run progresses.

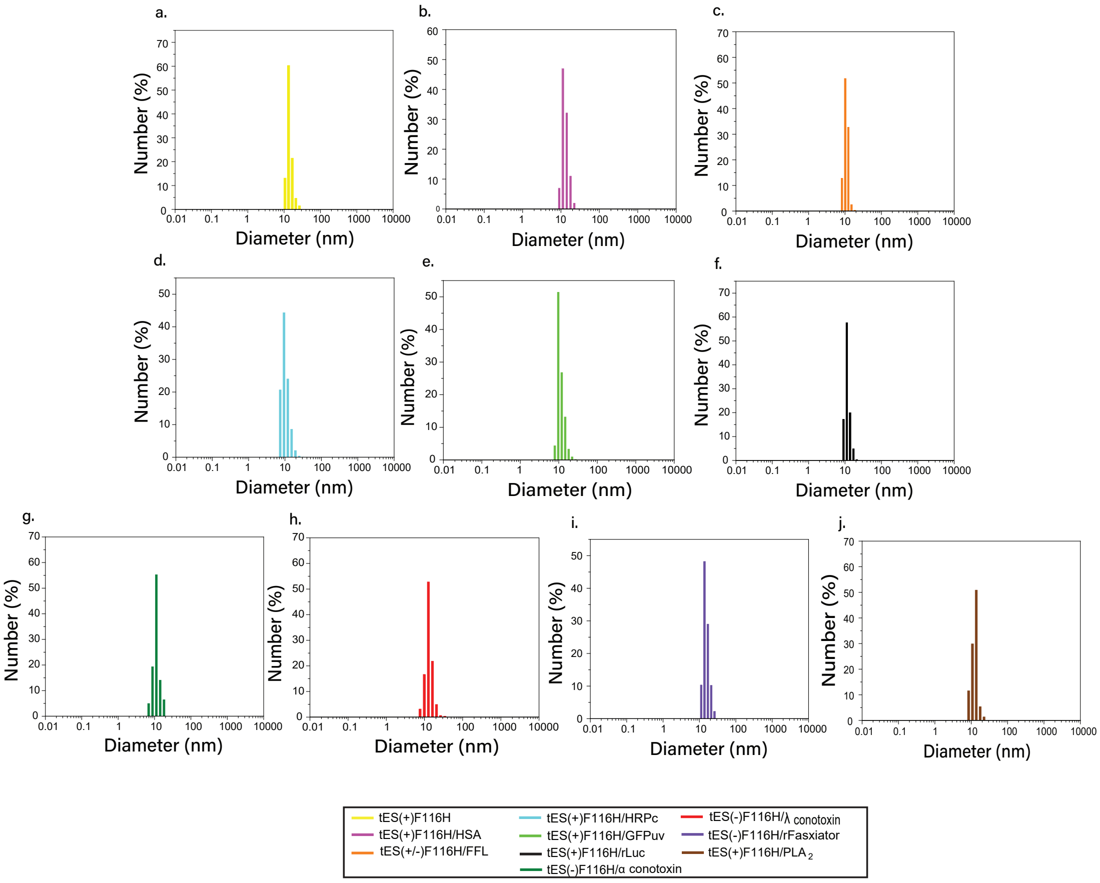

Supplementary Fig. 8 | Characterization of tES nanoparticles using Dynamic Light Scattering (DLS): DLS experiments showed no change in the hydrodynamic diameter (12 nm approx.) of POI encapsulated tES, suggesting internalization of POI within tES.

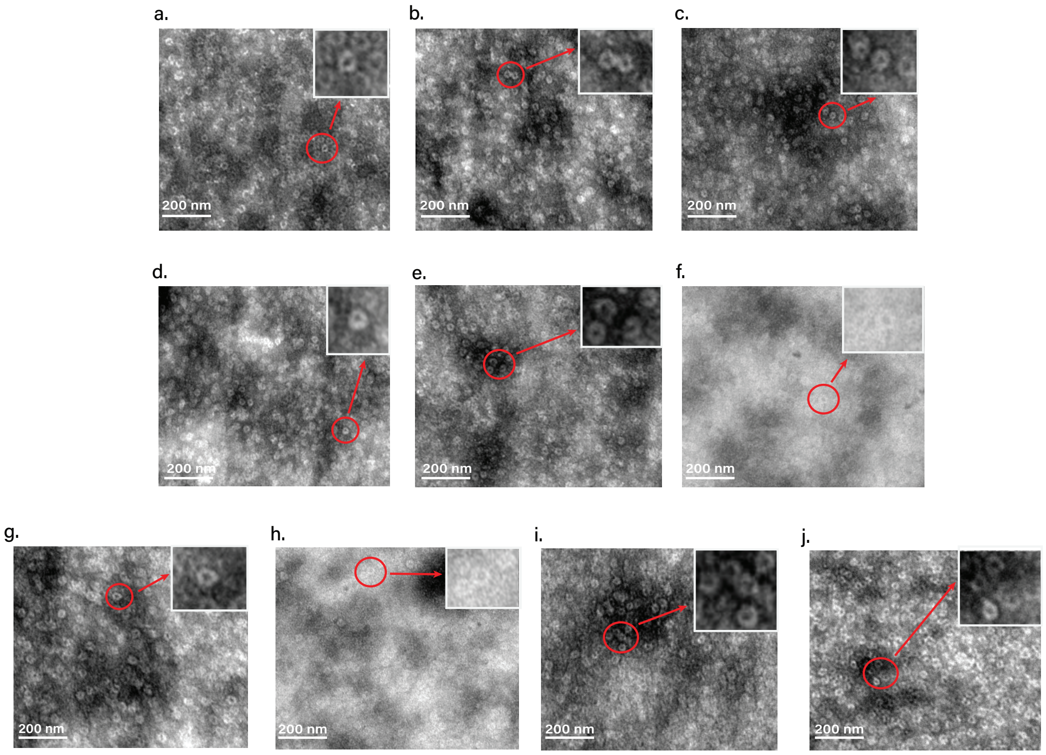

Supplementary Fig. 9 | Characterization of tES nanoparticles using Transmission Electron Microscopy (TEM): TEM experiments showed no change in the morphology and hydrodynamic diameter (12 nm approx.) of POI encapsulated tES, suggesting internalization of POI within tES. The scale bar is 200 nm (n=3 independent experiments).

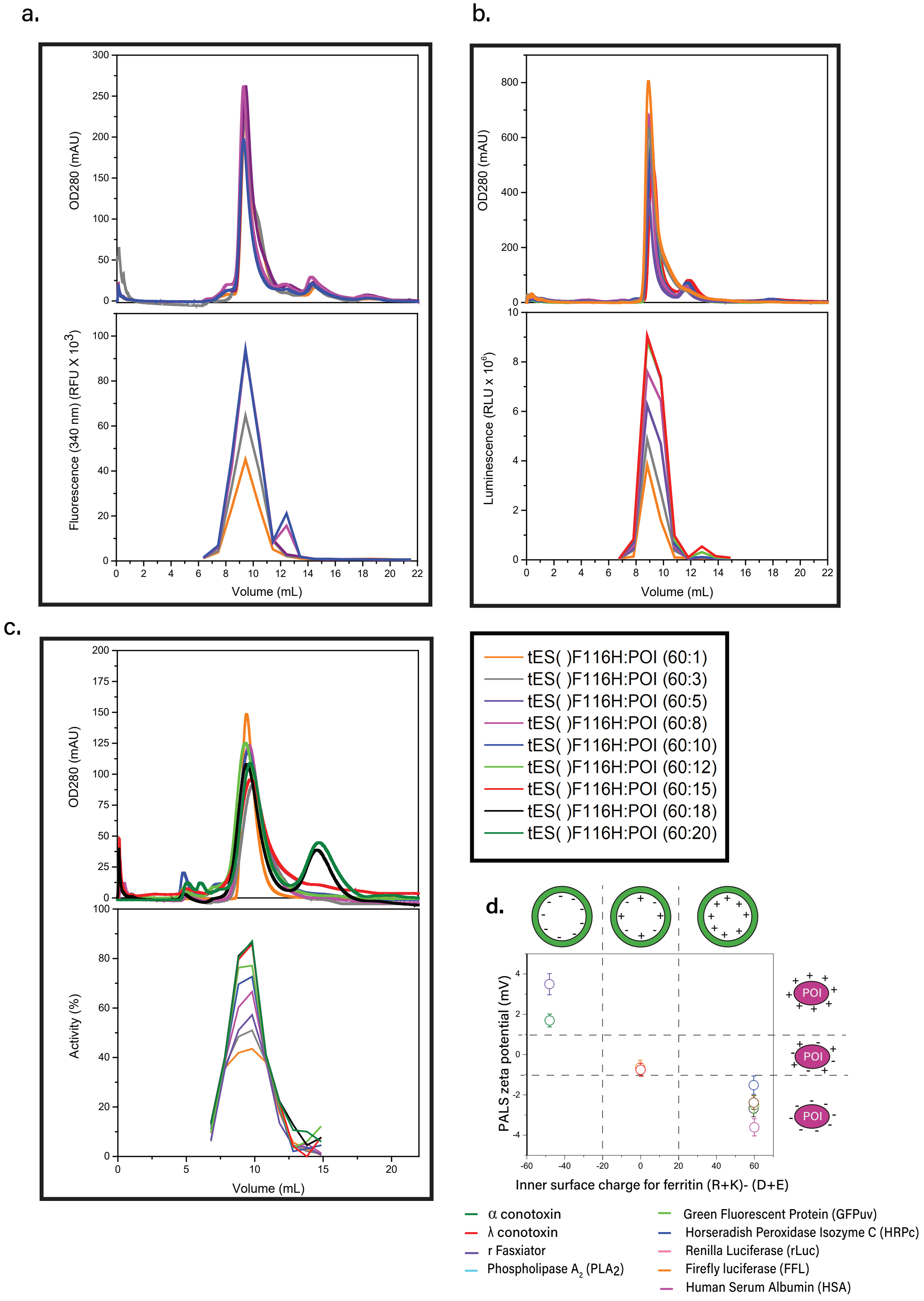

Supplementary Fig. 10 | Optimization of molar ratios of tES subunits to POI: Size-exclusion profiles (upper panel) of (a) tES-F116H(+):HSA (b) tES-F116H(+):rLuc (c) tES-F116H(-):rFasxiator with different molar ratios of tES subunits and POI. Each fraction of size-exclusion chromatography was analysed for POI activity for all molar ratios tested (lower panel). POI activity coincides with tES peak, suggesting its encapsulation inside tES assembly. All experiments were performed in triplicates, error bar represents ± standard deviation. (d) Charge matching of tES-F116H and the measured zeta potential of the POI predict optimal folding. Combinations with rank order highest functional yield are shown for nine monomeric POI's. tES-F116H(+), tES-F116H(+/-), tES-F116H(-) and POI-alone are indicated as (+), (+/-), (-) and h6, respectively (data are presented as mean ± SEM., n = 3 independent experiments).

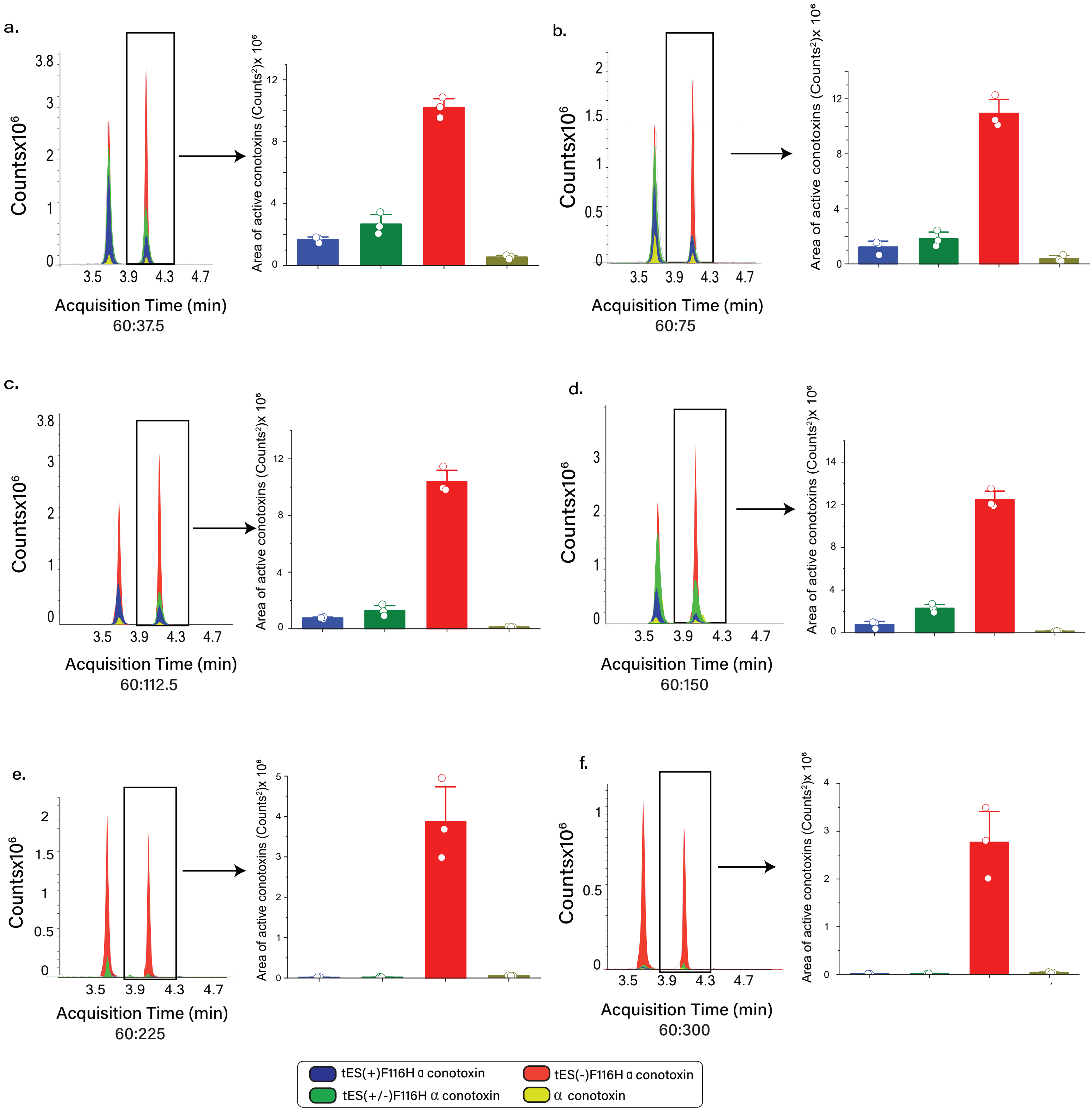

Supplementary Fig. 11 | Mass spectrometric analysis of α conotoxin: α conotoxin was folded in vitro without tES or within tES-F116H(+), tES-F116H(+/-) or tES-F116H(-) with different molar ratios of $tES_{subunits}$:α conotoxins from (a) 60:37.5, (b) 60:75, (c) 60:112.5, (d) 60:150, (e) 60:225 and (f) 60:300 respectively. Relative yield of globular (active) conformation when conotoxin is folded in presence and absence of tES is shown as a histogram
(data are presented as mean ± SEM., n = 3 independent experiments) (Source data are provided as a Source Data file).

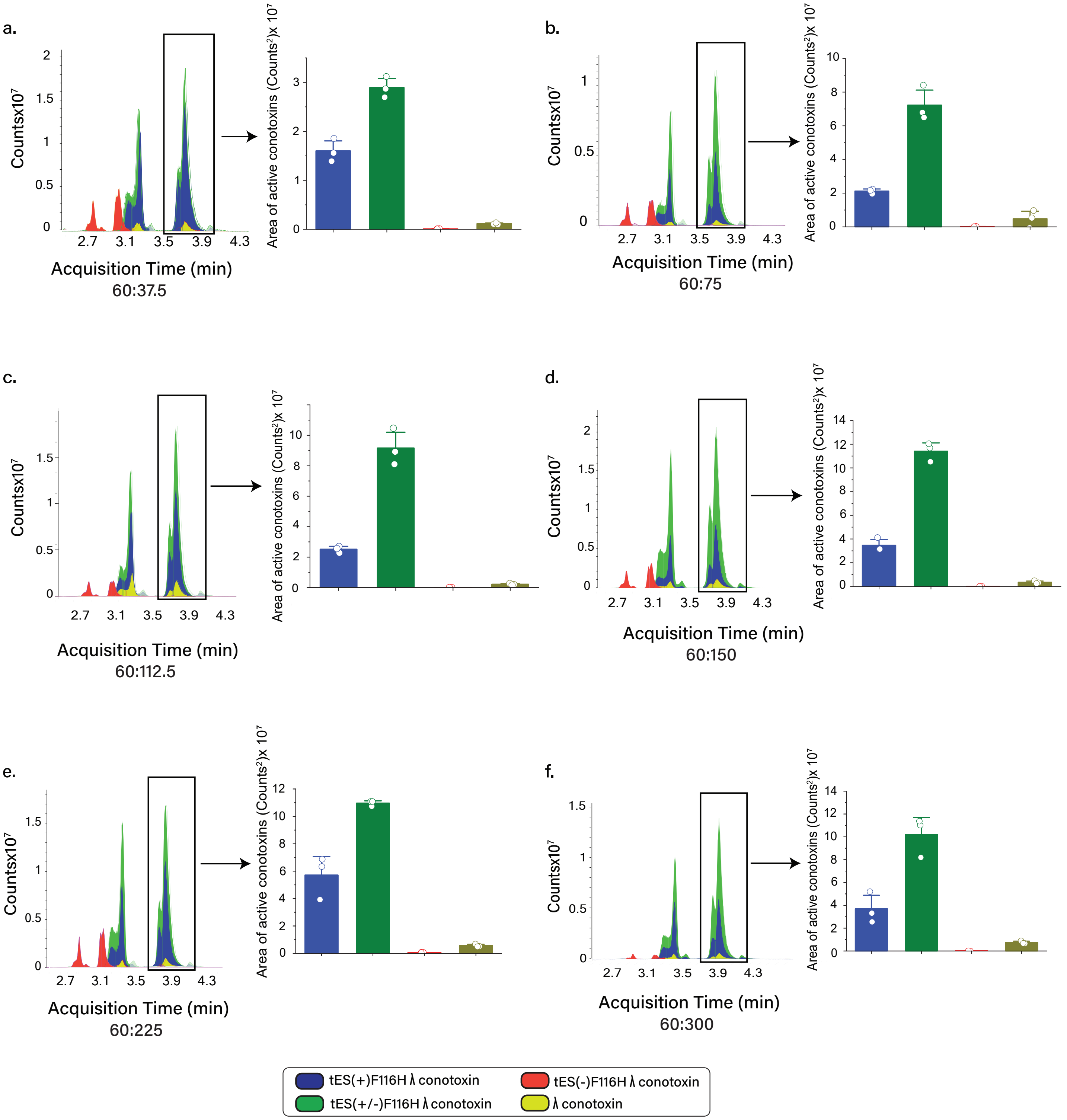

Supplementary Fig. 12 | Mass spectrometric analysis of λ conotoxin: λconotoxin was folded in vitro without tES or within tES-F116H(+), tES-F116H(+/-) or tES-F116H(-) with different molar ratios of $tES_{subunits}$:λ conotoxins from (a) 60:37.5, (b) 60:75, (c) 60:112.5, (d) 60:150, (e) 60:225 and (f) 60:300 respectively. Relative yield of globular (active) conformation when λ conotoxin is folded in presence and absence of tES is shown as a histogram (Data are presented as mean ± SEM., n = 3 independent experiments) (Source data are provided as a Source Data file).
