# Supplementary material for: A general approach to protein folding using thermostable exoshells
Source: Nat Commun. 2021 Sep 29;12:5720. doi: 10.1038/s41467-021-25996-4 (PMC8481291; doi:10.1038/s41467-021-25996-4)
Supplement: Supplementary file 2 — Reporting Summary [file 41467_2021_25996_MOESM2_ESM.pdf]

nature portfolio

Corresponding author(s): Chester L. Drum

Last updated by author(s): Sep 2, 2021

Reporting Summary

Nature Portfolio wishes to improve the reproducibility of the work that we publish. This form provides structure for consistency and transparency in reporting. For further information on Nature Portfolio policies, see our Editorial Policies and the Editorial Policy Checklist.

Statistics

For all statistical analyses, confirm that the following items are present in the figure legend, table legend, main text, or Methods section.

n/a	Confirmed	
☐	☒	The exact sample size (n) for each experimental group/condition, given as a discrete number and unit of measurement
☒	☐	A statement on whether measurements were taken from distinct samples or whether the same sample was measured repeatedly
☒	☐	The statistical test(s) used AND whether they are one- or two-sided<br>*Only common tests should be described solely by name; describe more complex techniques in the Methods section.*
☒	☐	A description of all covariates tested
☒	☐	A description of any assumptions or corrections, such as tests of normality and adjustment for multiple comparisons
☐	☒	A full description of the statistical parameters including central tendency (e.g. means) or other basic estimates (e.g. regression coefficient) AND variation (e.g. standard deviation) or associated estimates of uncertainty (e.g. confidence intervals)
☒	☐	For null hypothesis testing, the test statistic (e.g. F, t, r) with confidence intervals, effect sizes, degrees of freedom and P value noted<br>*Give P values as exact values whenever suitable.*
☒	☐	For Bayesian analysis, information on the choice of priors and Markov chain Monte Carlo settings
☒	☐	For hierarchical and complex designs, identification of the appropriate level for tests and full reporting of outcomes
☒	☐	Estimates of effect sizes (e.g. Cohen's d, Pearson's r), indicating how they were calculated

Our web collection on statistics for biologists contains articles on many of the points above.

Software and code

Policy information about availability of computer code

Data collection	No software was used
Data analysis	Clampfit 11.0.3 (Molecular Devices, LLC), Origin Lab 2018 (Northampton, MA, USA) (version 9.5), QuantStudio Real-Time PCR software (version 1.3) (Applied Biosystems), SEDFIT software (version 14.7g), SEDNTERP software (version 1.09).

For manuscripts utilizing custom algorithms or software that are central to the research but not yet described in published literature, software must be made available to editors and reviewers. We strongly encourage code deposition in a community repository (e.g. GitHub). See the Nature Portfolio guidelines for submitting code & software for further information.

Data

Policy information about availability of data

All manuscripts must include a data availability statement. This statement should provide the following information, where applicable:

- Accession codes, unique identifiers, or web links for publicly available datasets
- A description of any restrictions on data availability
- For clinical datasets or third party data, please ensure that the statement adheres to our policy

Data sharing not applicable to this article as no datasets were generated or analysed during the current study.

Field-specific reporting

Please select the one below that is the best fit for your research. If you are not sure, read the appropriate sections before making your selection.

- [x] Life sciences
- [ ] Behavioural & social sciences
- [ ] Ecological, evolutionary & environmental sciences

For a reference copy of the document with all sections, see nature.com/documents/nr-reporting-summary-flat.pdf

Life sciences study design

All studies must disclose on these points even when the disclosure is negative.

Sample size	12.<br>In the present study, the selected 12 proteins cover a broad range of substrates that differ in their size (1 - 66 kDa), isoelectric point (4 - 8), and number of disulfide bonds (0 - 17). Moreover, these proteins vary in their secondary structure and exist as either monomers or multimers.
Data exclusions	No data were excluded
Replication	The experiments were tested at least in triplicates and all attempts at replication were successful.
Randomization	Not applicable, We selected the proteins to represent a broad substrate category. Moreover, some of the samples like p53 and small toxins were suggested by our collaborators
Blinding	We were blinded to group allocation during data collection and/or analysis. Different monomeric or polymeric proteins with different sizes were selected. The We had no prior knowledge of which protein(s) would show promising results.

Reporting for specific materials, systems and methods

We require information from authors about some types of materials, experimental systems and methods used in many studies. Here, indicate whether each material, system or method listed is relevant to your study. If you are not sure if a list item applies to your research, read the appropriate section before selecting a response.

Materials & experimental systems

n/a	Involved in the study	
[ ]	[x]	Antibodies
[x]	[ ]	Eukaryotic cell lines
[x]	[ ]	Palaeontology and archaeology
[x]	[ ]	Animals and other organisms
[x]	[ ]	Human research participants
[x]	[ ]	Clinical data
[x]	[ ]	Dual use research of concern

Methods

n/a	Involved in the study	
[x]	[ ]	ChIP-seq
[x]	[ ]	Flow cytometry
[x]	[ ]	MRI-based neuroimaging

Antibodies

Antibodies used	mouse monoclonal anti-p53 antibody clone PAb421 (SIGMA, MABE283), mouse monoclonal anti-p53 (wild type) antibody clone PAb1620 (SIGMA, MABE339), mouse monoclonal antip53 antibody Pab240 (Abcam, ab26), mouse monoclonal anti-HA (hemagglutinin) antibody (SIGMA, SAB3500873).
Validation	mouse monoclonal anti-HA antibody:<br>Application by supplier: Immunoblotting, immunocytochemistry, immunoprecipitation<br>Application by other sources:<br>• Kryszke M-H, Adjeriou B, Liang F, Chen H, Dautry F. Post-transcriptional gene silencing activity of human GIGYF2. Biochemical and biophysical research communications. 2016;475(3):289-94.<br>• Zhang Y, Feng Y, Xin Y, Liu X. SGIP1 dimerizes via intermolecular disulfide bond in µHD domain during cellular endocytosis. Biochemical and biophysical research communications. 2018;505(1):99-105.<br>Species reactivity statement by supplier: Recognizes the human influenza virus hemagglutinin (HA), known as the HA tag<br><br>mouse monoclonal anti p53 antibody, Pab240<br>Application by supplier: Immunoblotting (Western Blotting), Immunofluorescence, Immunoprecipitation, Paraffin Sections, Flow Cytometry, Frozen Sections, Gel Shift<br>Conformational flexibility of mutant p53 was indicated by the conformation-dependent epitope recognized by PAB240 clone (Genetic and immunochemical analysis of mutant p53 in human breast cancer cell lines AND Milner, J. Flexibility: the key to p53 function? Trends Biochem. Sci. 20, 49-51 (1995) AND Gannon J, Greaves R, Iggo R, Lane D. Activating mutations in p53 produce a common

conformational effect. A monoclonal antibody specific for the mutant form. The EMBO journal. 1990;9(5):1595-602.). This clone has been used from that time abundantly for different applications including Immunoblotting, Immunofluorescence, Immunoprecipitation, etc. Some references are listed below:

- Wei G, Liu G, Liu X. Identification of two serine residues important for p53 DNA binding and protein stability. FEBS letters. 2003;543 (1-3):16-20.
- Hailfinger S, Jaworski M, Marx-Stoelting P, Wanke I, Schwarz M. Regulation of P53 stability in p53 mutated human and mouse hepatoma cells. International journal of cancer. 2007;120(7):1459-64.
- Nishio K, Inoue A. Senescence-associated alterations of cytoskeleton: extraordinary production of vimentin that anchors cytoplasmic p53 in senescent human fibroblasts. Histochemistry and cell biology. 2005;123(3):263-73.
- Delverdier M, Albaric O, Bret L, Amardeihl M. Immunohistochemical expression of p53 in animal tumors: a methodological study using four anti-human p53 antibodies. Histology and histopathology. 2001;16(1):113-21.

Species reactivity statement by supplier: Bovine, Chicken, Hamster, Human, Mouse, Rat
